# Pericytes take up and degrade α-synuclein but succumb to apoptosis under cellular stress

**DOI:** 10.1038/s41598-022-20261-0

**Published:** 2022-10-15

**Authors:** Taylor J. Stevenson, Rebecca H. Johnson, Jimmy Savistchenko, Justin Rustenhoven, Zoe Woolf, Leon C. D. Smyth, Helen C. Murray, Richard L. M. Faull, Jason Correia, Patrick Schweder, Peter Heppner, Clinton Turner, Ronald Melki, Birger V. Dieriks, Maurice A. Curtis, Michael Dragunow

**Affiliations:** 1 Department of Pharmacology, University of Auckland, Private Bag 92019, Auckland, 1142 New Zealand; 2 Centre for Brain Research, University of Auckland, Private Bag 920139, Auckland, 1142 New Zealand; 3 Alternative Energies and Atomic Energy Commission and Laboratory of Neurodegenerative Diseases, Molecular Imaging Research Center, Francois Jacob Institute, National Center for Scientific Research, Fontenay-Aux-Roses, France; 4 Department of Anatomy and Medical Imaging, University of Auckland, Private Bag 92019, Auckland, 1142 New Zealand; 5 Auckland City Hospital, 2 Park Road, Auckland, 1010 New Zealand; 6 Starship Children’s Hospital, 2 Park Road, Auckland, 1010 New Zealand; 7 Department of Anatomical Pathology, Lab Plus, Auckland City Hospital, 2 Park Road, Auckland, New Zealand

**Keywords:** Blood-brain barrier, Cell death in the nervous system, Cellular neuroscience, Molecular neuroscience, Stress and resilience, Parkinson's disease

## Abstract

Parkinson’s disease (PD) is characterised by the progressive loss of midbrain dopaminergic neurons and the presence of aggregated α-synuclein (α-syn). Pericytes and microglia, two non-neuronal cells contain α-syn in the human brain, however, their role in disease processes is poorly understood. Pericytes, found surrounding the capillaries in the brain are important for maintaining the blood–brain barrier, controlling blood flow and mediating inflammation. In this study, primary human brain pericytes and microglia were exposed to two different α-synuclein aggregates. Inflammatory responses were assessed using immunocytochemistry, cytometric bead arrays and proteome profiler cytokine array kits. Fixed flow cytometry was used to investigate the uptake and subsequent degradation of α-syn in pericytes. We found that the two α-syn aggregates are devoid of inflammatory and cytotoxic actions on human brain derived pericytes and microglia. Although α-syn did not induce an inflammatory response, pericytes efficiently take up and degrade α-syn through the lysosomal pathway but not the ubiquitin–proteasome system. Furthermore, when pericytes were exposed the ubiquitin proteasome inhibitor—MG132 and α-syn aggregates, there was profound cytotoxicity through the production of reactive oxygen species resulting in apoptosis. These results suggest that the observed accumulation of α-syn in pericytes in human PD brains likely plays a role in PD pathogenesis, perhaps by causing cerebrovascular instability, under conditions of cellular stress.

## Introduction

Parkinson’s disease (PD) is a multifaceted disease characterised by the presence of abnormal α-synuclein (α-syn) in the form of Lewy bodies and Lewy neurites^1^. The pathogenesis of PD is not well understood, however, the main processes involved include inflammation, altered protein turnover and oxidative stress^2–5^. We recently discovered that non-neuronal cells such as pericytes, microglia and astrocytes contain α-syn in the human brain^6^, however, their involvement in disease processes are poorly understood.

Neuroinflammation has gained significant traction as a main contributor to the pathogenesis of PD in recent years. Several cytokines and chemokines including tumour necrosis factor α (TNF-α), Interleukin-1β (IL-1β) and monocyte chemoattractant protein 1 (MCP-1) are present in the brains, cerebrospinal fluid (CSF) and blood of PD patients^7–9^. Several studies show that extracellular monomeric, oligomeric and aggregated α-syn induces a pro-inflammatory response in rodent microglia through the release of cytokines increased cyclooxygenase-2 (COX-2) and inducible nitric oxide synthase (iNOS) as well as the production of free radicals^10–19^. Brain pericytes surround endothelial cells of brain capillaries forming the neurovascular unit where they maintain the integrity of the blood–brain barrier and the control of blood flow^20–22^. Pericytes also have the ability to transfer the α-syn protein to other pericytes in vitro^23^*.* Importantly, pericytes are one of the first brain resident cells to respond to systemic inflammation^21,24,25^ and can be activated by monomeric α-syn in rat pericytes in vitro^26^ and in a mouse model overexpressing human α-syn^27^. However, the inflammatory role of α-syn in human brain derived pericytes is yet to be elucidated.

The presence of accumulated α-syn in sporadic PD begs the question of whether there is a dysregulation of the mechanisms that control the levels of α-syn in the brain^28^. The ubiquitin proteasome system (UPS) and the autophagy lysosomal pathway (ALP) are considered the two main pathways for protein degradation, however, the direct mechanism through which α-syn is degraded remains controversial^29–31^. Pericytes have a significant number of lysosomes and are capable of degrading phagocytosed material and could be involved in the uptake and degradation of α-syn in the human brain^21^.

α-syn mediated cell toxicity may be related to the increased production of reactive oxygen species (ROS) and modifications in the mitochondrial electron transport chain^4^. A reduction in complex I activity is evident, leading to ROS production in the substantia nigra of patients with sporadic PD^4,32^. Interestingly, ROS influences both the UPS and ALP systems, impairing their ability to eliminate damaged proteins, ultimately leading to further protein misfolding and ultimately cell death^33^. Evidence of increased ROS and impaired α-syn degradation in pericytes and other non-neuronal cells is lacking.

In this study, we investigated the effects of α-syn on human brain-derived pericytes. We demonstrated that α-syn does not induce an inflammatory response in primary human brain pericytes or microglia after the addition of two structurally different fibrillar α-syn polymorphs. However, we confirm that primary human brain pericytes can efficiently take up and subsequently degrade α-syn aggregates in vitro and that degradation is regulated through the ALP. Although α-syn alone did not induce cell death or inflammation, in combination with another stressor (the UPS inhibitor MG132), it resulted in significant cell death. The two hit or multiple hit hypothesis suggests that two or more insults are required to cause PD^34–36^. These results support the multiple hit hypothesis in PD and highlight the importance of pericytes within the blood–brain barrier in the pathogenesis of PD.

## Results

### Inflammatory responses in pericytes and microglia

**Figure 1:**
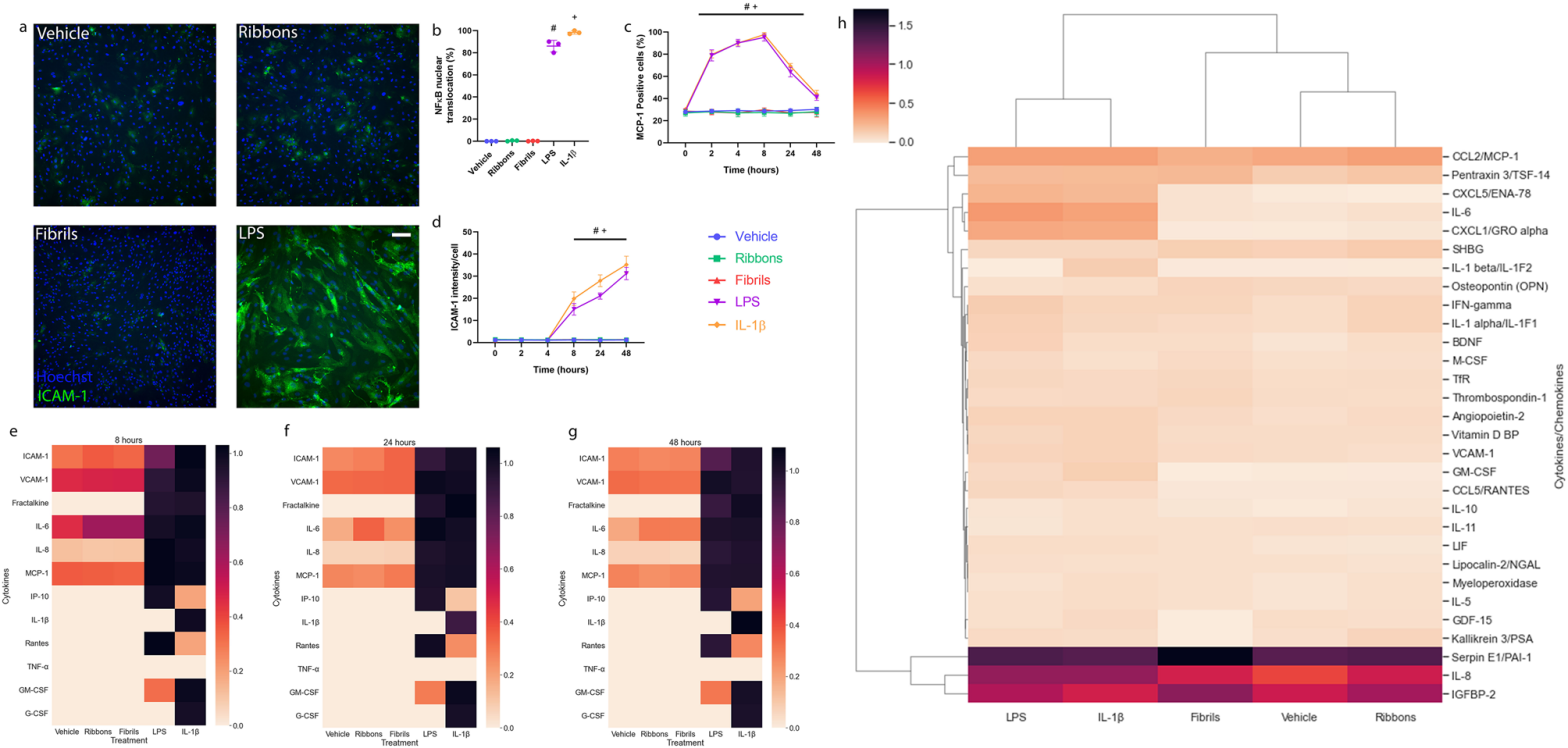
Inflammatory responses of pericytes treated with two α-syn preparations. Inflammatory responses of primary human brain pericytes when treated with vehicle, ribbons, fibrils, (100 nM), LPS (10 ng/mL) and IL-1β (10 ng/mL). (**a**) Representative immunocytochemistry images of ICAM-1 induction after 48 h treatment. Scale bar = 200 µm. Immunocytochemistry quantification of (**b**) NF-κB nuclear translocation after 1 h treatment, (**c**) MCP-1 and (**d**) ICAM-1 induction after times indicated (*n* = 3 epilepsy cases*, mean* ± *SD*). Pericytes were cultured and treated with vehicle, two α-syn preparations (100 nM), LPS (10 ng/mL) and IL-1β (10 ng/mL) before conditioned media were taken and cytokine secretion analysed by cytometric bead array. Heatmap analysis of cytokine secretion of pericytes after treatment at (**e**) 8 h, (**f**) 24 h and (**g**) 48 h (*n* = 3 epilepsy cases). Pericytes were cultured and treated with vehicle, two different α-synuclein preparations (100 nM), LPS (10 ng/mL) and IL-1β (10 ng/mL) for 24 h before conditioned media were taken and cytokine secretion measured using a Proteome Profiler Human XL Cytokine Array Kit. Clustered heatmap analysis (**h**) of the top 30 secretions in pericytes (*n* = 1, epilepsy case). # LPS, + IL-1β vs vehicle *p* < 0.05; one way ANOVA with Tukey’s multiple comparisons test or two way ANOVA with Tukey’s multiple comparisons test.

**Figure 2:**
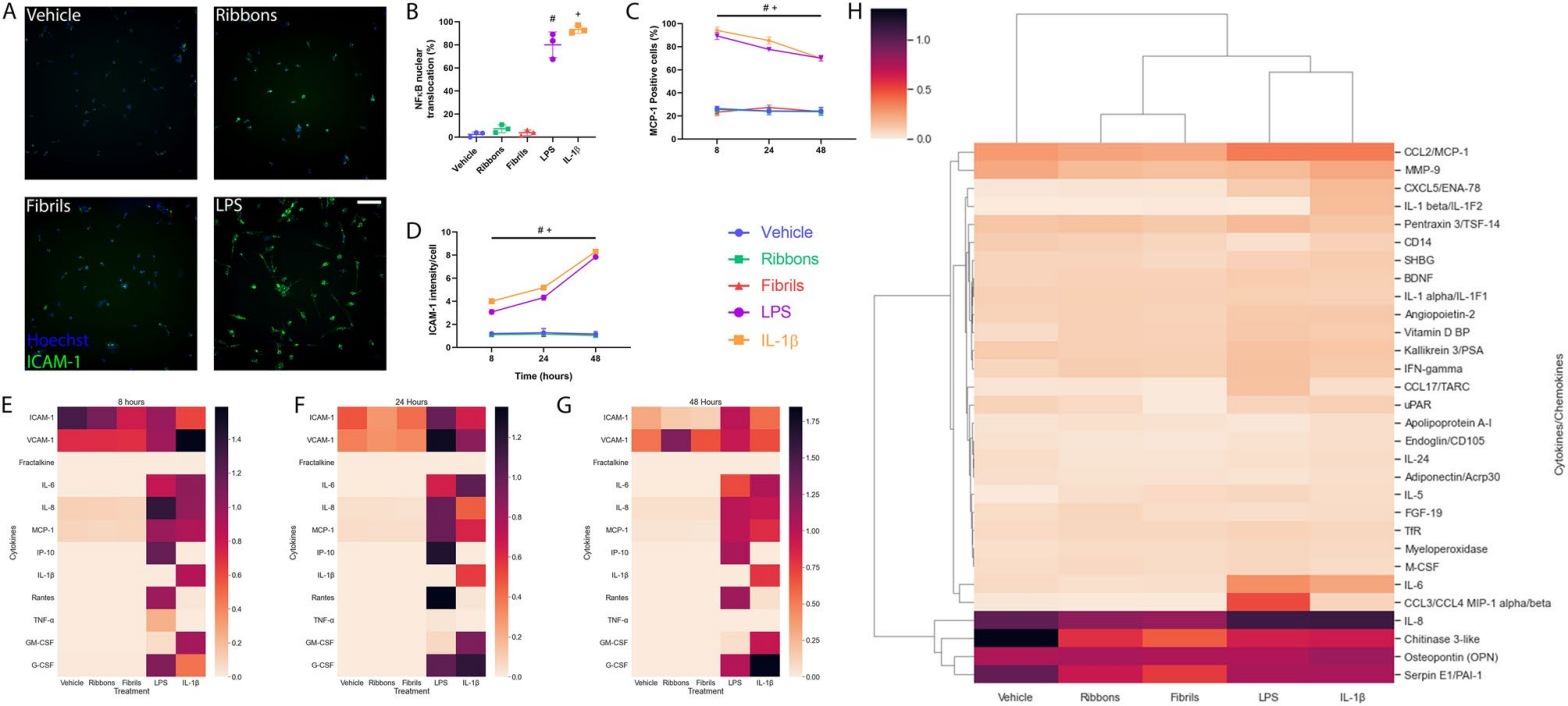
Inflammatory responses of microglia treated with two α-syn preparations. Inflammatory responses of primary human brain microglia when treated with vehicle, ribbons, fibrils (100 nM), LPS (10 ng/mL) and IL-1β (10 ng/mL). (**A**) Representative immunocytochemistry images of ICAM-1 induction after 48 h treatment Scale bar = 200 µm. Immunocytochemistry quantification of (**B**) NF-κB nuclear translocation after 1 h treatment, (**C**) MCP-1 and (**D**) ICAM-1 induction after times indicated (*n* = 3, *mean* ± *SD*). Microglia were cultured and treated with vehicle, two different α-synuclein preparations (100 nM), LPS (10 ng/mL) and IL-1β (10 ng/mL) before conditioned media were taken and cytokine secretion analysed by cytometric bead array. Heatmap analysis of cytokine secretion of microglia after treatment at (**E**) 8 h, (F) 24 h and (**G**) 48 h (*n* = 3). Microglia were cultured and treated with vehicle, two different α-synuclein preparations (100 nM), LPS (10 ng/mL) and IL-1β (10 ng/mL) for 24 h before conditioned media were taken and cytokine secretion measured using a Proteome Profiler Human XL Cytokine Array Kit. Clustered heatmap analysis (**H**) of the top 30 secretions in microglia (*n* = 1, epilepsy case). #LPS, + IL-1β vs vehicle *p* < 0.05; one way ANOVA with Tukey’s multiple comparisons test or two way ANOVA with Tukey’s multiple comparisons test.

Pericytes and microglia were treated with two different α-syn polymorphs—ribbons and fibrils (Table 1). The ribbons and fibrils did not induce the expression of the adhesion molecule intracellular adhesion molecule-1 (ICAM-1) (Figs. 1a,d, 2a–d), nuclear factor kappa-light-chain-enhancer of activated B cells (NF-κB) nuclear translocation after 1 h treatment (Figs. 1b, 2b) or increase MCP-1 expression (Figs. 1c, 2c) in pericytes or microglia while the positive control inflammatory stimuli—LPS and IL-1β did (Figs. 1a–d, 2a–d). None of the treatments induced cell death (Supplementary Fig. 1).

**Table 1:** α-Synuclein and cytokine treatments.

Compounds	Concentration	Supplier/previous publications	Catalogue
IL-1β	10 ng/mL	PeproTech, NJ, USA	200-01B
LPS (*E. coli*, 026:B6)	10 ng/mL	Sigma, MO, USA	L4391
Ribbons	100 nM	Ghee et al.^73^, Bousset et al.^72^, Gath et al.^74^, Peelaerts et al.^69^, Makky et al.^75^ and Verasdonck et al.^76^ rPeptide, GA, USA rPeptide, GA, USA	–
Fibrils	100 nM		–

Conditioned media were collected from pericytes and microglia treated with the two α-syn aggregates, LPS and IL-1β and the cell secretions were analysed using a cytometric bead array (CBA). Heatmap analysis of pericyte secretions at 8, 24 and 48 h (Figs. 1e–g, 2e-g) reveal that both the ribbons and fibrils do not induce an inflammatory response in either pericytes or microglia (Supplementary Figs. 2, 3).

Lastly, conditioned media were taken from pericytes at 24 h and cytokine secretions were analysed using a Proteome Profiler Human XL Cytokine Array Kit. Supporting the CBA and ICC data, the two α-syn aggregates did not induce the expression of any cytokines or chemokines in pericytes or microglia (Figs. 1h, 2h; Supplementary Figs. 4, 5). Additionally, concentrations of α-syn from 15 nM up to 1 µM did not induce significant inflammatory responses in human brain pericytes (Supplementary Fig. 6).

**Figure 3:**
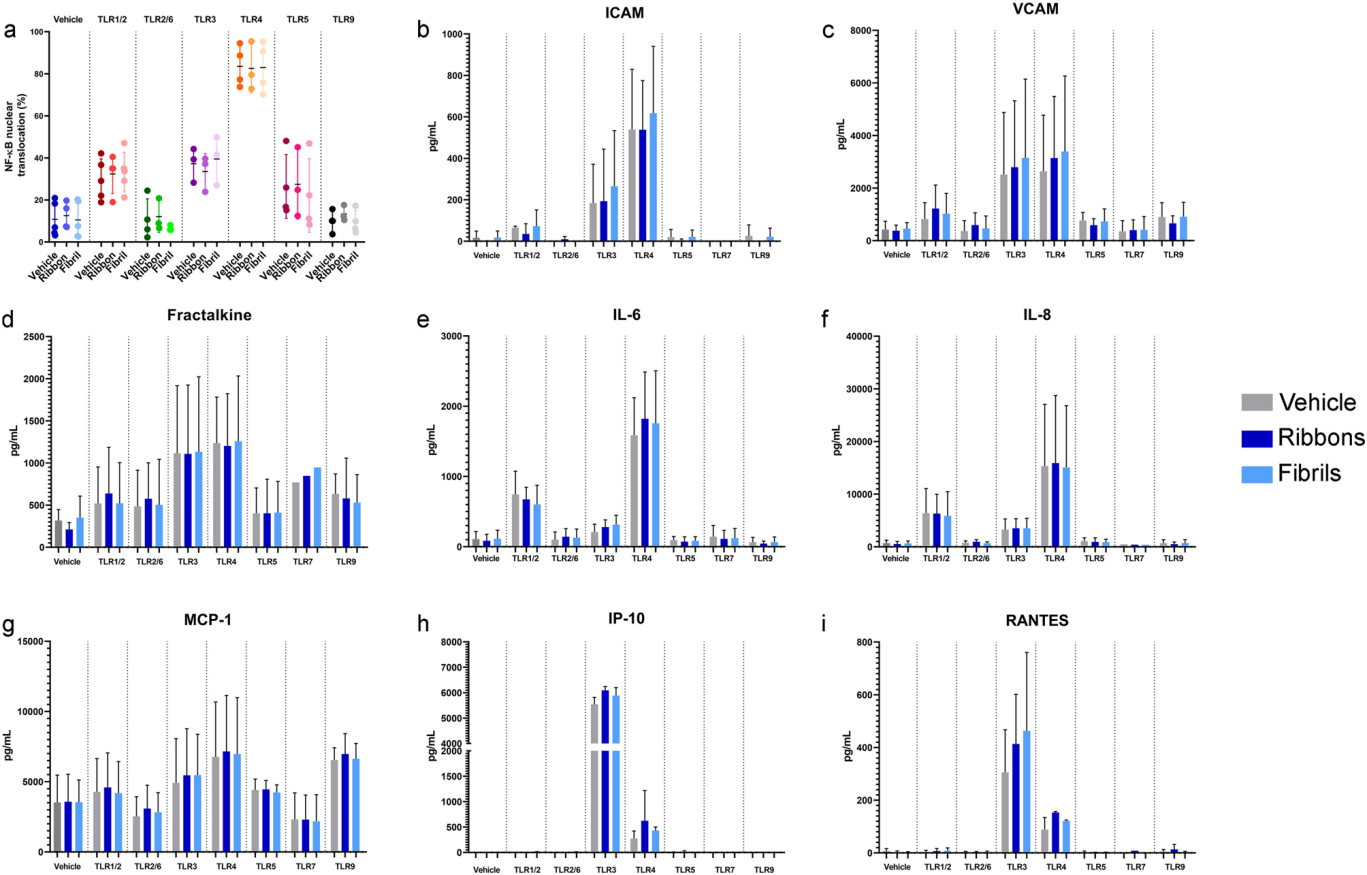
TLR ligands and α-syn treatment on pericytes. Inflammatory responses of primary human brain pericytes when treated with vehicle, ribbons (100 nM), fibrils (100 nM) and TLR ligands (Supplementary Table S5). Immunocytochemistry quantification of (**a**) NF-κB nuclear translocation after 1 h treatment. Conditioned media were taken and cytokine secretion analysed by cytometric bead array after 24 h. Secretions of (**b**) ICAM-1, (**c**) VCAM-1, (**d**) Fractalkine, (**e**) IL-6, (**f**) IL-8, (**g**) MCP-1, (**h**) IP-10 and (**i**) RANTES at 24 h after treatment (*n* = 5 epilepsy cases, mean ± SD).

Pericytes responded to several TLR ligands (Supplementary Table 6), such as Pam3SCK4 (TLR1/2), Poly (I:C) (TLR3), LPS (TLR4), Flagellin (TLR5) and CpG ODN (TLR9). However, the ribbons and fibrils did not induce additional NF-κB nuclear translocation (Fig. 3a) or trigger additional release of pro-inflammatory cytokines when treated in sequence with the TLR ligands compared to treatment with TLR ligands alone (Fig. 3b–i). These data suggest that α-syn did not affect TLR ligand signalling in pericytes. Conditioned media collected from pericytes and microglia after 24 h of co-treatment of LPS with ribbons or fibrils showed no significant changes in the amount of pro-inflammatory cytokines released compared to LPS alone (Supplementary Figs. 7, 8).

Microglia were included as a comparison for the inflammatory responses. The remaining results were only completed on pericytes.

### Pericytes take up and degrade α-synuclein

**Figure 4:**
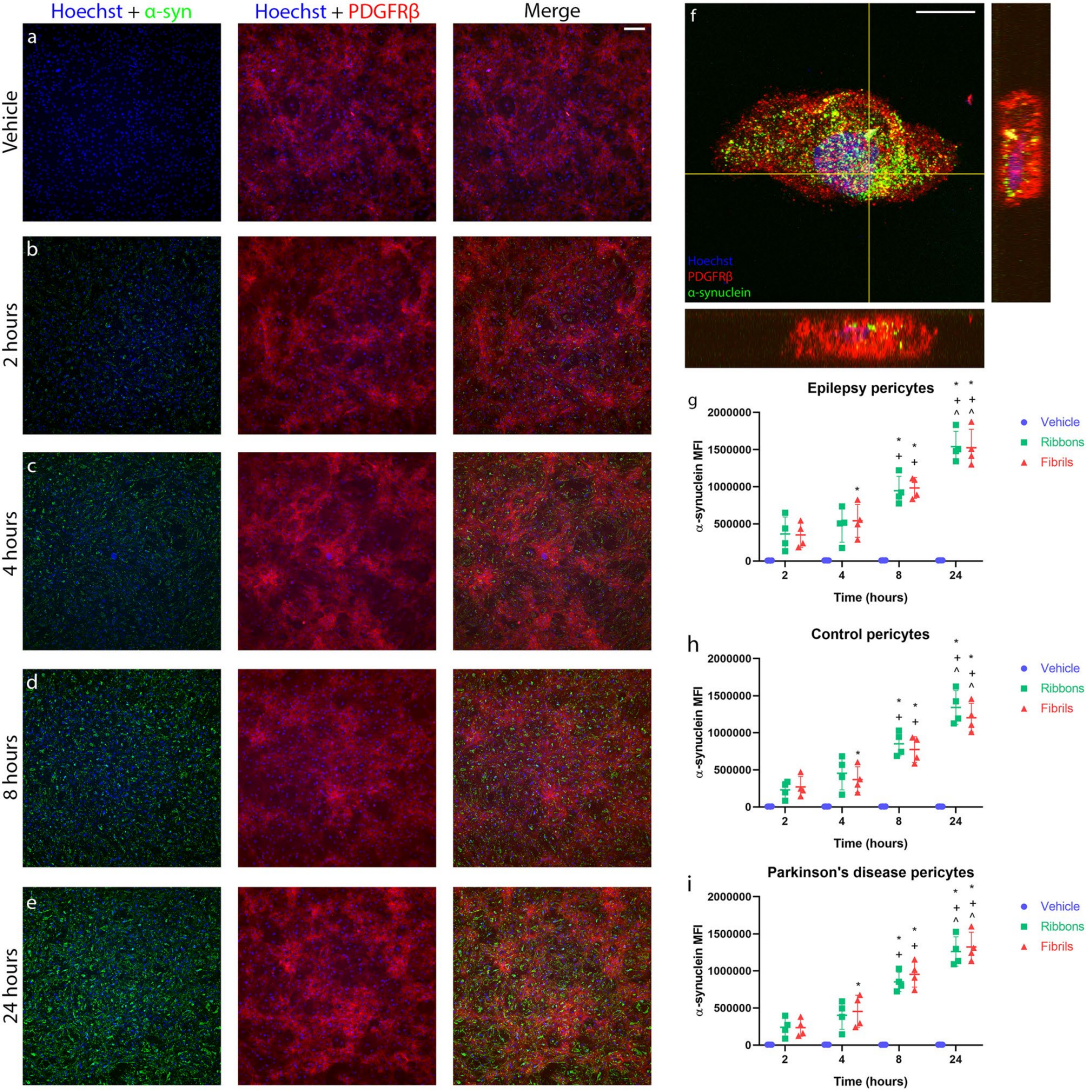
Uptake of α-syn in primary human brain pericytes. Pericytes were incubated with α-syn fibrils (100 nM) for 24 h. Pericytes did not contain endogenous α-syn (**a**), however, pericytes efficiently take up α-syn at (**b**) 2 h, (**c**) 4 h and (**d**) 8 h and (**e**) 24 h. (**F**) Orthogonal confocal microscopy reveals that α-syn is present within pericytes in vitro. Scale bar, 100 µm (**a–e**), Scale bar 10 µm (**f**). α-syn MFI was measured using fixed-flow cytometry in (**g**) epilepsy pericytes. (**h**) control pericytes and (**i**) PD pericytes after incubation with ribbons (100 nM) or fibrils (100 nM) for 24 h. (*n* = 4 in each group, mean ± SD). *p < 0.05 compared to 2 h treatment, ^*p* < 0.05 compared to 4 h treatment, ^*p* < 0.05 compared to 8 h treatment; two way ANOVA with Tukey’s multiple comparisons test.

No endogenous α-syn was detected in the primary human brain pericytes in culture (Fig. 4a). Following the addition of ribbons (100 nM) or fibrils (100 nM) into the culture medium, pericytes began to take up the α-syn within 2 h (Fig. 4b–d). Maximal uptake was observed at 24 h after addition (Fig. 4e). Confocal microscopy confirmed the internalisation of α-syn in pericytes in vitro (Fig. 4f). Pericytes were treated with cytochalasin D, an inhibitor of actin polymerisation or colchicine, a microtubule disruptor and uptake was partially blocked (Supplementary Fig. 9). Fixed flow cytometry was used to quantify intracellular α-syn (Supplementary Fig. 10) and showed a significant increase in mean fluorescent intensity (MFI) of α-syn as the incubation time increased in epilepsy, control and PD post-mortem pericytes (Fig. 4g–i). There were no significant differences in the uptake of the two α-syn aggregates between the different groups of pericytes (Supplementary Fig. 11).

**Figure 5:**
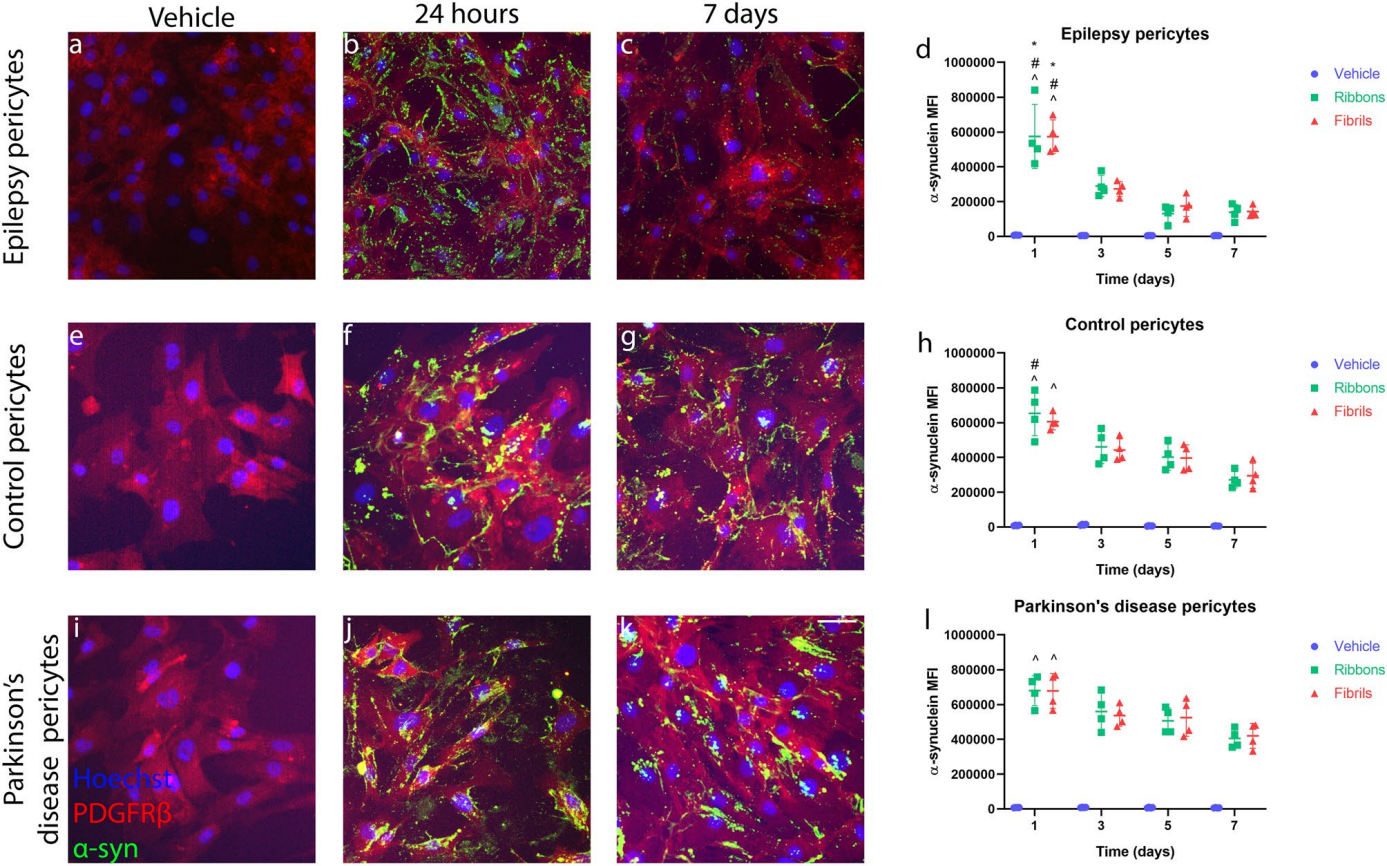
Degradation of α-syn in primary human brain pericytes. Immunocytochemistry images of pericytes incubated with α-syn fibrils (50 nM) for (**a**) vehicle, (**b**) 24 h, (**c**) 7 days in epilepsy pericytes. (**d**) α-syn MFI was measured using fixed flow cytometry with ribbons (50 nM) or fibrils (50 nM) over 7 days in epilepsy pericytes. Post-mortem control pericytes were exposed to α-syn fibrils (50 nM) for (**e**) vehicle, (**f**) 24 h, (**g**) 7 days in epilepsy pericytes. (**h**) α-syn MFI was measured using fixed flow cytometry with ribbons (50 nM) or fibrils (50 nM) over 7 days in control pericytes. Post-mortem PD pericytes were exposed to α-syn fibrils (50 nM) for (**i**) vehicle, (**j**) 24 h, (**k**) 7 days Scale bar = 10 µm. (**l**) α-syn MFI was measured using fixed flow cytometry with ribbons (50 nM) or fibrils (50 nM) over 7 days in control pericytes. Scale bar = 10 µm. (*n* = 4 in each group, mean ± SD). *p < 0.05 compared to 3 day treatment, ^#^*p* < 0.05 compared to 5 day treatment, ^*p* < 0.05 compared to 7 day treatment; two way ANOVA with Tukey’s multiple comparisons test.

Qualitative immunocytochemistry showed that biopsy derived epilepsy pericytes degraded ribbons (50 nM) and fibrils (50 nM) (Fig. 5a–c), where the majority of α-syn has been removed after 7 days of incubation (Fig. 5c,d). Quantification using fixed flow cytometry showed there was a significant decrease in α-syn MFI from the 1 day time point compared to all the time points indicated for both α-syn aggregates (Fig. 5d). After day 3 there were no significant differences in the MFI compared to day 5 and 7. On the contrary, post-mortem derived control and PD pericytes showed less degradation of α-syn after 7 days of incubation compared to epilepsy pericytes (Fig. 5e–l; Supplementary Fig. 11). Control pericytes showed a significant decrease in α-syn MFI from day 1 compared to day 5 and 7 for the ribbons, and for the fibrils, a significant decrease between day 1 and 7. For the PD pericytes, both the ribbons and fibrils only saw a significant decrease in α-syn between day 1 and day 7. There were no significant differences in degradation between the control and PD pericytes (Supplementary Fig. 11).

**Figure 6:**
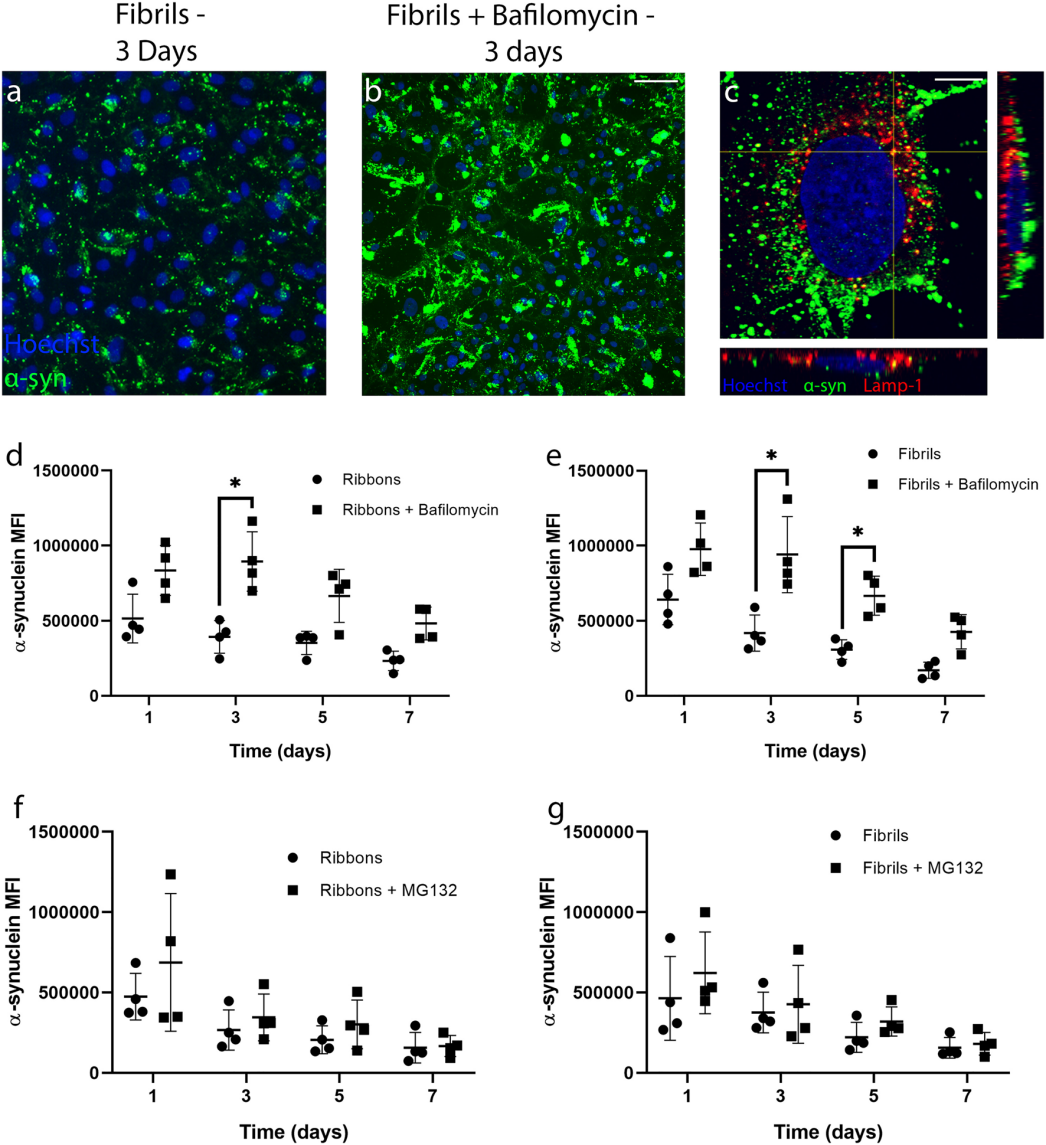
Mechanisms of degradation of α-syn in primary human brain pericytes. Pericytes were incubated with (**a**) α-syn alone for 3 days or (**b**) pre-treated with bafilomycin (200 nM). (**c**) Confocal microscopy with orthogonal views of α-syn co-localising with LAMP-1 vesicles. Scale bar = 10 µm. Pericytes were pre-treated with bafilomycin (200 nM) for 4–6 h prior to a 7 days incubation and MFI was measured using fixed flow cytometry for (**d**) ribbons (50 nM) or (**e**) fibrils (50 nM). Pericytes were pre-treated with MG132 (5 µM) for 6–8 h prior to a 7 days incubation and MFI was measured using fixed flow cytometry for (**f**) ribbons (50 nM) or (**g**) fibrils (50 nM). (*n* = 4 in each group, mean ± SD). *p < 0.05.

To investigate the mechanism of degradation, pericytes were pre-treated with bafilomycin for 4–6 h before incubation with α-syn. Immunocytochemistry showed a significant increase in α-syn fibrils present when pre-treated with bafilomycin compared with α-syn fibrils treatment alone (Fig. 6a,b). Confocal microscopy showed that intracellular α-syn partially co-localised with LAMP-1 positive lysosomes in primary human brain pericytes (Fig. 6c). Bafilomycin inhibited the degradation of α-syn in pericytes as demonstrated by a significant increase in α-syn MFI measured after 3 days of incubation with both the ribbons and fibrils and 5 days of incubation with the fibrils (Fig. 6d,e). There were no significant differences at the other time points investigated.

To investigate whether the UPS was involved in α-syn degradation by pericytes, we first tested the UPS inhibitor MG132 alone and found that it caused significant cell death in both a concentration and time dependent manner (Supplementary Fig. 12). Treatment with 5 µM MG132 for 6 h, however, did not induce significant pericyte death and therefore, this concentration was used to investigate α-syn degradation. Following treatment with MG132 for 6 h, pericytes were incubated with either ribbons or fibrils for 7 days, however, no significant differences in α-syn levels were seen between the different time points (Fig. 6f,g).

**Figure 7:**
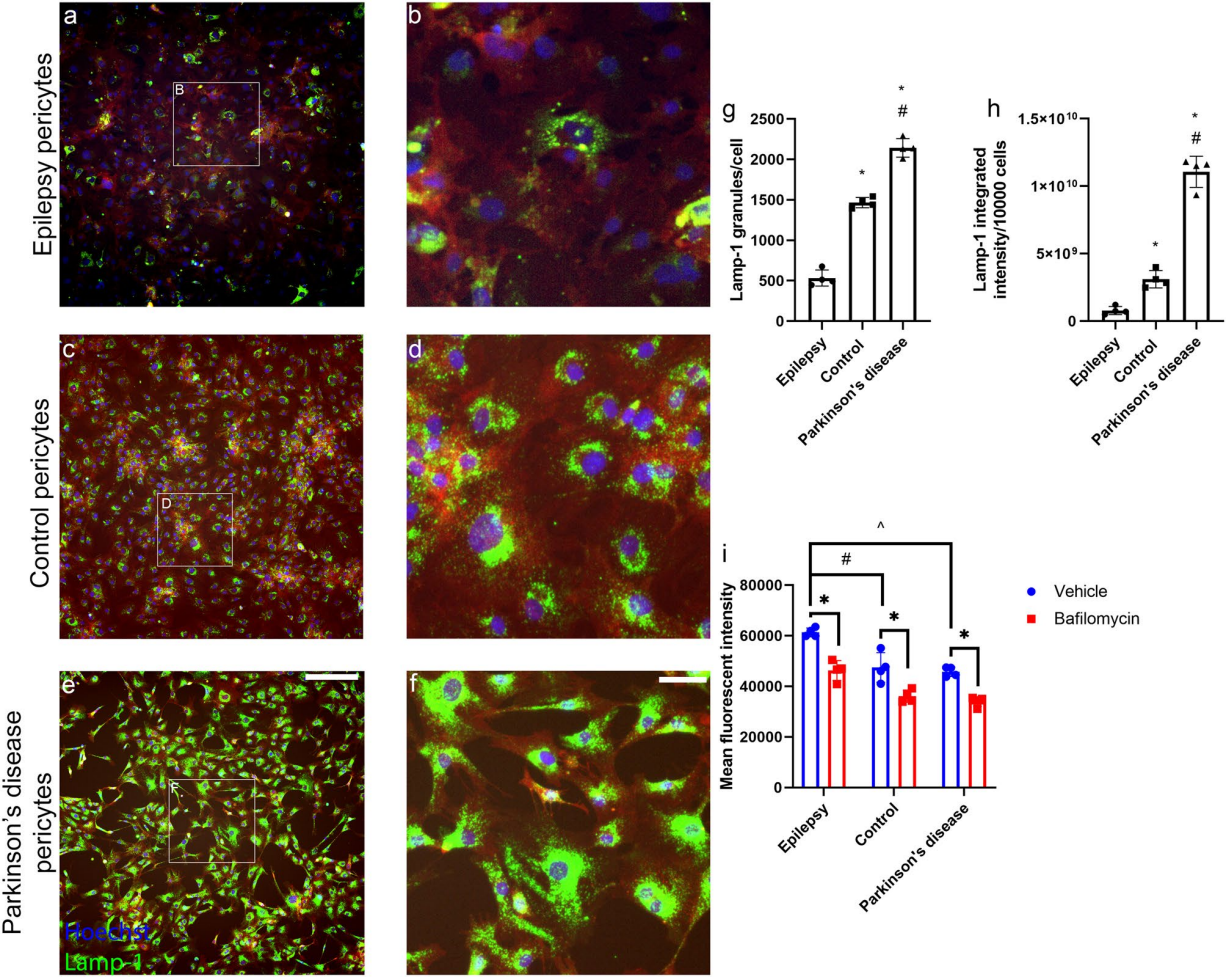
Lysosomes in epilepsy, control and Parkinson’s disease pericytes. Primary human brain pericytes were stained with the LAMP-1 lysosomal antibody to investigate differences in (**a,b**) epilepsy, (**c,d**) control and (**e,f**) PD pericytes. Scale bar = 100 µm (**a,c,e**) and 40 µm (**b,d,f**). LAMP-1 was quantified via immunocytochemistry, (**g**) number of LAMP-1 granules per cell were quantified in epilepsy, control and PD pericytes. (**h**) LAMP-1 integrated intensity per 10,000 cells was quantified in epilepsy, control and PD pericytes*.* (*n* = 4 in each group, mean ± SD). *p < 0.05 compared to epilepsy, ^#^*p* < 0.05 compared to control. Primary human brain pericytes were pre-treated with bafilomycin for 4–6 h and the lysosome function was measured (**i**) using live flow cytometry to measure the MFI after treatment with vehicle or bafilomycin. (*n* = 4 in each group, mean ± SD). *p < 0.05 compared to vehicle and bafilomycin in their respective groups, ^#^*p* < 0.05 vehicle epilepsy compared to vehicle control, ^*p* < 0.05 vehicle epilepsy compared to vehicle.

These results show that the ALP, but not the UPS system is involved in α-syn degradation by pericytes. Interestingly, we found a stark difference in the intensity and number of lysosomes between the different groups of pericytes (Fig. 7a–h). Epilepsy pericytes, had the lowest number of LAMP-1 positive granules and intensity, while PD pericytes had the highest (Fig. 7g,h). Interestingly, control and PD pericytes had the least functional lysosomes, while epilepsy pericyte’s lysosomes were the most functional as per the lysosomal activity assay (Fig. 7i).

### Pericyte cell death

**Figure 8:**
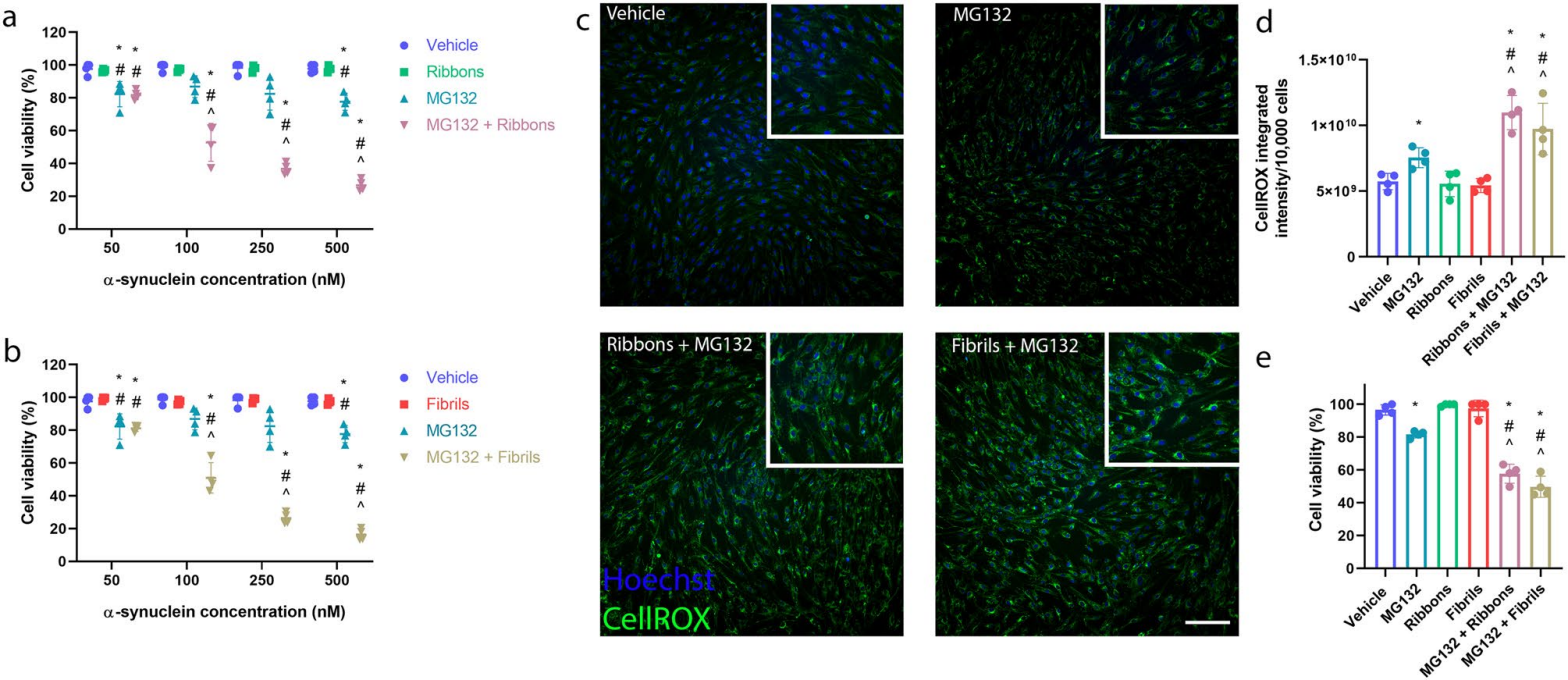
Primary human brain pericytes response to α-syn pre-treated with MG132. Cell viability of primary human brain pericytes when pre-treated with MG132 (5 µM) for 6 h prior to the incubation with either (**a**) ribbons (50–500 nM) or (**b**) fibrils (50–500 nM) for 24 h*.* (*n* = 4 epilepsy cases, mean ± SD). *p < 0.05 compared to vehicle, ^#^*p* < 0.05 compared to either ribbons or fibrils in the respective graph, ^*p* < 0.05 compared to MG132 treatment alone; two way ANOVA with Tukey’s multiple comparisons test. (**c**) Representative immunocytochemistry images of the ROS production using cellROX in primary human brain pericytes after treatment with vehicle, MG132, MG132 and ribbons and MG132 and fibrils. Scale bar = 50 µm. (**d**) Graphs showing the amount of ROS produced using cellROX integrated intensity when normalized to 10,000 cells when pre-treated with 5 µM MG132 for 6 h prior to co-incubation with either ribbons (100 nM) or fibrils (100 nM) for 24 h. (*n* = 4 epilepsy cases, mean ± SD). (**e**) Graphs showing cell viability of primary human brain pericytes when pre-treated with 5 µM MG132 for 6 h prior to co-incubation with either ribbons (100 nM) or fibrils (100 nM) for 24 h*.* *p < 0.05 compared to vehicle, ^#^*p* < 0.05 compared to MG132 treatment, ^*p* < 0.05 compared to ribbons or fibrils in the respective graph, ^+^*p* < 0.05 compared to MG132 or ribbons and fibrils treatment in the respective graph; one way ANOVA with Tukey’s multiple comparisons test.

As described above, α-syn alone at 100 or 500 nM at both 24 h and 48 h timepoints had no effect on cell viability (Supplementary Fig. 1) and MG132 alone at 5 µM for 6 h also did not induce significant cell death (Supplementary Fig. 12). However, cell death was induced in a concentration dependent manner with a combination of α-syn and MG132. When 50 nM of either ribbons (Fig. 8a) or fibrils (Fig. 8b) were added to pericytes pre-treated with MG132, there were no significant differences compared to MG132 treatment alone. However, at α-syn concentrations of 100 nM and above, significant differences were seen between co-treatment of MG132 with either ribbons or fibrils compared with each treatment alone (Fig. 8a,b). Therefore, 100 nM of α-syn was chosen for subsequent experiments as this resulted in significant cell death in the presence of MG132. No cellular toxicity was observed when co-treated with bafilomycin (Supplementary Fig. 13).

Live cell imaging showed that MG132 by itself induces ROS production in pericytes compared to vehicle (Fig. 7c). Pre-treatment with MG132 and subsequent incubation with either ribbons or fibrils significantly increased the amount of ROS production in pericytes (Fig. 8c). This is further reflected through the increased integrated intensity of the cellROX fluorogenic dye (Fig. 8d). The significant increase in ROS also coincides with the decrease in cell viability (Fig. 8e).

**Figure 9:**
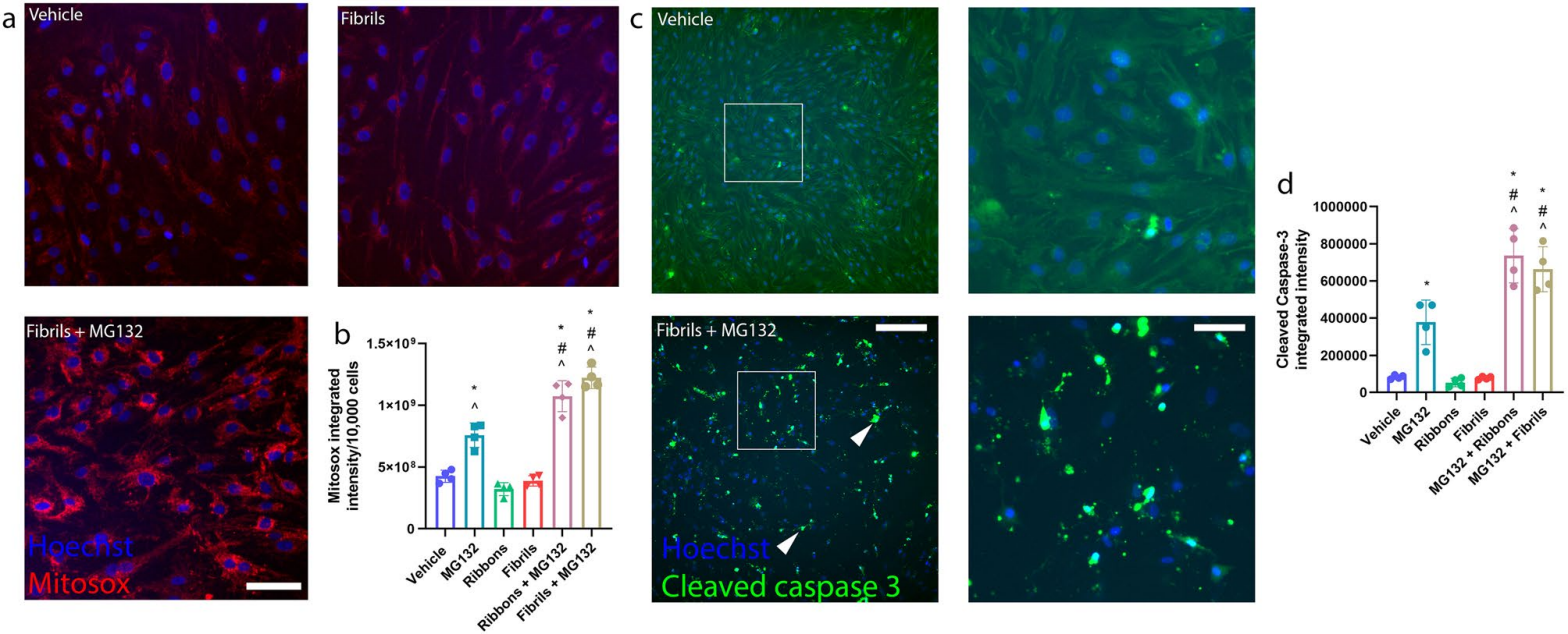
Cell death of primary human brain pericytes in response to pre-treatment with MG132 and α-syn. (**a**) Representative immunocytochemistry images of MitoSOX staining in primary human brain pericytes after treatment with vehicle, α-syn fibrils (100 nM), MG132 (5 µM) pre-treatment for 6 h following α-syn fibrils (100 nM) incubation and (**b**) graph showing the integrated intensity of MitoSOX after MG132 (5 µM) pre-treatment for 6 h following α-syn fibrils (100 nM) incubation. Scale bar = 50 µm. (*n* = 4 epilepsy cases, mean ± SD). *p < 0.05 compared to vehicle, ^*p* < 0.05 compared to ribbons or fibrils, ^#^*p* < 0.05 compared to MG132; one way ANOVA with Tukey’s multiple comparisons test. (**c**) Representative immunocytochemistry images of cleaved-caspase 3 staining in primary human brain pericytes pre-treated with 5 µM MG132 for 6 h prior to incubation vehicle or MG132 and fibrils. Scale bar = 100 µm. (**d**) Graph showing cleaved-caspase 3 integrated intensity after pericytes were pre-treated with 5 µM MG132 prior to co-incubation with either ribbons (100 nM) or fibrils (100 nM). (*n* = 4 epilepsy cases, mean ± SD). *p < 0.05 compared to vehicle, ^#^*p* < 0.05 compared to MG132 treatment, ^*p* < 0.05 compared to either ribbon or fibrils; one way ANOVA with Tukey’s multiple comparisons test.

When pericytes were treated with fibrils or ribbons alone there were no significant differences in the production of superoxide compared to vehicle (Fig. 9a,b). However, in the presence of MG132, significant differences were seen in superoxide production by mitochondria compared to α-syn or MG132 alone (Fig. 9b).

From qualitative immunocytochemistry, pericytes that were pre-treated with MG132 and α-syn had a significant increase in cleaved-caspase 3 positive staining compared to vehicle (Fig. 9c). Immunocytochemical quantification further strengthened this observation demonstrating that there are significant increases in the integrated intensity of cleaved-caspase 3 staining in pericytes that were treated with MG132 and either ribbons or fibrils compared to vehicle and MG132 treatment alone (Fig. 9d).

## Discussion

Our results show that α-syn fibrillar polymorphs alone did not elicit inflammatory and cytotoxic actions on human brain derived pericytes and microglia. Pericytes, however, efficiently take up and degrade aggregated α-syn through the lysosomal pathway, but not through the UPS pathway. Although inhibiting the UPS with MG132 had no effect on α-syn degradation in pericytes, the combination of MG132 with α-syn was cytotoxic through the generation of ROS and caspase-3 mediated apoptosis. Our results suggest that pericytes might normally take up and degrade extracellular α-syn aggregates even at high concentrations but when they are exposed to other stressors involved in PD the accumulated α-syn may become toxic causing pericyte loss and cerebrovascular instability.

In this study we found that aggregated α-syn did not trigger an inflammatory response. This observed lack of effect in human brain derived pericytes and microglia is inconsistent with previous reports^16,37–41^ (see Supplementary Table 7). Human recombinant α-syn protein applied at various concentrations has been used in many rodent models and non-brain derived human models in vitro to study inflammation^16,26,37–41^. In fact, little work has been completed on primary human brain cells. In this study, we show that α-syn may not act as an inflammatory agent in pericytes and microglia. This is in contrast with a recent study showing that the same fibrils used in this study, induced a significant inflammatory response in human peripheral blood monocytes and the mouse BV-2 microglial cell line^40^. These results, pose interesting questions about whether human non-brain derived cells such as peripheral immune cells, even those isolated from PD patients, or IPSCs, react differently to α-syn compared to brain resident immune cells.

Previous studies have demonstrated that α-syn is able to directly activate rodent microglia through the classical activation pathway that also includes the upregulation of TLRs such as TLR2, TLR3 and TLR4^15,16,42^. Therefore, we investigated whether α-syn may interact with TLRs and their various ligands in primary human brain pericytes. It has been documented that LPS activates TLR4 in pericytes, altering the structure of these cells and inducing inflammation^43,44^. However, we found that α-syn ribbons and fibrils do not synergise with the TLR ligands and therefore do not alter inflammatory responses through TLRs in pericytes (Fig. 3).

Intestinal infection by bacteria may lead to PD-like symptoms and increase the amount of pathogenic α-syn in the gut^45,46^. Furthermore, small amounts of endotoxins in recombinant α-syn can induce an inflammatory response that is greater than what would be expected from the endotoxins alone in peripheral blood mononuclear cells^37^. Therefore, we investigated whether LPS together with aggregated α-syn may produce a greater inflammatory response by potentially activating a pathogen-associated molecular pattern (PAMP) or damage-associated molecular pattern (DAMP) responses. We found no alteration in the inflammatory responses in both pericytes upon co-treatment with LPS and the two α-syn aggregates (Supplementary Figs. 7, 8).

Although these α-syn aggregates did not induce an inflammatory response in human brain resident pericytes, these cells are involved in other disease processes. Interestingly, we have shown that pericytes contain α-syn in the human olfactory bulb^6^, however, we now confirm that pericytes can actively take up α-syn from the extracellular space (Fig. 5; Supplementary Fig. 9) and, therefore could be actively involved in disease processes by taking up aggregated α-syn from the brain parenchyma. Pericytes have been previously shown to have phagocytic capabilities, where they act as the last line of defence in the BBB, cleaning up the extracellular space and degrading foreign protein and debris^47,48^.

Pericytes possess many lysosomal granules suggesting that they are equipped to degrade components that are phagocytosed and endocytosed^49–51^. Significant degradation of aggregated α-syn is likely to occur through the lysosomal pathways of chaperone-mediated autophagy (CMA) and macroautophagy where dysfunction of these systems may contribute to PD pathogenesis^52^. We found that bafilomycin which inhibits the acidification of the lysosomes^53,54^ caused the accumulation of α-syn aggregates in primary human brain pericytes and delayed the degradation of both α-syn ribbons and fibrils (Fig. 6d,e). These results suggest that the likely pathway for aggregated α-syn degradation in pericytes in vitro is the ALP. The difference in the number of lysosomes present and their functional characteristics between the different groups of pericytes provides evidence that the degradation pathways may be altered (Fig. 7). PD pericytes had the highest number of lysosomes compared to control and epilepsy pericytes. They were however the least effective at degrading aggregated α-syn. The marked increase in the number of lysosomes may be a form of compensation due to lysosomal impairment^55^. Indeed, many mutations found in PD have been linked to the ALP pathway including leucine-rich repeat kinase-2 (LRRK2), parkin and phosphatase and tensin homolog-induced putative kinase 1 (PINK1). Additionally, genes associated with lysosomal dysfunction have also been linked to PD such as glucocerebrosidase (GBA) and lysosomal type 5 P-type ATPase (ATP13A2)^55^. Therefore, extensive evidence suggests that lysosomal dysfunction may lead to the subsequent accumulation of aggregated α-syn.

Other studies have shown that both the 20S and 26S proteasomes degrade specific species of α-syn such as monomers and low molecular weight oligomers^3,29,52,56^. The inhibition of the UPS with MG132 in this study did not impair the ability of pericytes to degrade α-syn aggregates (Fig. 6f,g). Therefore, this pathway is likely not involved in the degradation of larger molecular weight, ribbons and fibrils in pericytes used in this study.

The differences in the ability of biopsy versus post-mortem pericytes to degrade α-syn may be caused by a number of factors including disease state, age of the cases and post-mortem delay. One of the main contributing factors for the difference in the degradation ability of control and PD pericytes may be that the activity of the UPS and ALP systems tends to decrease with age^52^. On average, the epilepsy pericytes used in this study were 30 years old, whereas control cases were 61 years old and PD cases 77.5 years old (Supplementary Tables 1, 3, 4). These differences can be seen in both the rate at which they degrade α-syn (Fig. 5) and the lysosomal functionality (Fig. 7). Another contributing factor could be the fact that the control and PD pericytes are taken from post-mortem brains. In comparison the epilepsy pericytes are taken from surgical biopsies. Therefore, there could be inherent differences between biopsy and post-mortem cultured cells. The PD pericytes had an average post-mortem delay of 4.5 h whereas the control pericytes had an average of 17.6 h post-mortem delay. These delays in time of culturing the cells could impact their function and could contribute to their decreased ability to degrade α-syn. Of course, epilepsy derived pericytes are unlikely to be “normal” so there is also a possibility that the differences between epilepsy and post-mortem control pericytes is due to the underlying epileptic condition or the residual effects of seizures.

MG132 is a potent inhibitor of the 26S proteasome complex^57^. Previous studies have shown pericyte loss in the PD brain^58^ increased string vessel formation^59^ and BBB disruption^59–61^. These changes might be mediated by the actions of α-syn on pericytes. α-syn aggregates did not induce inflammation (Fig. 1) or cell toxicity (Supplementary Fig. 1) alone, however, in the presence of another stressor, significant cell death was observed (Fig. 7a,b). The results from this study suggest that a double hit is toxic to pericytes exposed to aggregated α-syn.

The presence of ROS further exacerbates pericyte death. Pericytes may be particularly susceptible to ROS production. MG132 can induce ROS and decrease glutathione (GSH) in human pulmonary fibroblast cells leading to cell death^62^. Additionally, the majority of the ROS may be produced by the mitochondria in the form of superoxide in pericytes after exposure to MG132 and aggregated α-syn (Fig. 8a,b). Superoxide is mainly released from complexes I and III of the electron transport chain, suggesting that under these conditions, a significant amount of cellular oxidative stress occurs^63^.

Pericyte cell death may also in part relate to their inability to cope with the α-syn aggregates. Transformed cells including cancer cells, which accumulate misfolded, mutated or damaged proteins, are much more susceptible to proteasome inhibition than normal cells^62,64,65^. Adding extracellular α-syn aggregates to primary human pericytes may be overloading the protein quality control mechanisms. This is further demonstrated with the aggregated α-syn concentration response upon pre-treatment with MG132 (Fig. 7a,b). Additionally, it has been demonstrated that various proteasome inhibitors including MG132, can initiate apoptotic cell death through the induction of ROS^62,66^. ROS formation and subsequent GSH depletion by proteasome inhibitors may also trigger mitochondrial dysfunction leading to programmed apoptotic cell death^67,68^. Indeed, pre-treatment with MG132 and α-syn ribbons and fibrils leads to an increase in apoptotic cell death through the increased staining of cleaved-caspase 3 (Fig. 8c,d). Interestingly, bafilomycin co-treatment with α-syn in pericytes did not result in cellular toxicity or the production of ROS, although it resulted in α-syn build-up in the pericytes (Fig. 6; Supplementary Fig. 13). This suggests that the variability of pericytes, while sensitive to UPS inhibition, is not affected by inhibition of ALP. Therefore, our results suggest that an additional insult is required together with α-syn accumulation to induce pericyte death. This has important ramifications for the understanding of cell death in PD in the human brain.

Lastly, we show that there were no significant differences between the two conformational strains of α-syn in pericytes in inflammation, uptake and degradation in pericytes. Previous work demonstrated that different strains elicit different responses. Fibrils are the major toxic strain and result in motor impairments and cell death in vivo*,* while ribbons cause distinct histopathological phenotypes similar to multiple system atrophy^69^. Different strains of α-syn also spread, seed and target neuronal cells differently when injected into the olfactory bulb of wild-type mice^70^. The lack of differential effects seen in pericytes may in part be due to their role in the removal of α-syn rather than seeding and aggregation. Pericytes had no endogenous α-syn, therefore the cytotoxic effects thought to be associated with seeding and aggregation may not be present. Further in vivo studies tracking the spread of α-syn in non-neuronal cells will be important for determining whether these different strains display unique characteristics and whether they can seed and aggregate in non-neuronal cells like pericytes in the brain.

This study examined the effects of aggregated α-syn on pericytes to understand its role in PD pathogenesis. Our results demonstrate that aggregated α-syn does not induce an inflammatory response in primary human brain pericytes and microglia. Primary human brain pericytes can efficiently take up and subsequently degrade aggregated α-syn, although post-mortem control and PD pericytes are slightly impaired. The degradation of α-syn aggregates in pericytes has important implications for understanding how this protein may accumulate in the brain and provides a potential therapeutic target that could be exploited further to decrease the α-syn aggregate burden. Our results also demonstrate that α-syn aggregates exacerbate other cellular insults to pericytes. This expands our understanding of the mechanism by which α-syn aggregates may contribute to cell dysfunction in PD suggesting that a double hit may be required to induce pericyte cell death in the brain.

## Methods

### Tissue sources

The use of human tissue and collection procedures were approved by the Northern Regional Ethics Committee (New Zealand) for biopsy tissue (AKL/88/025). Biopsy human brain tissue was obtained with informed written consent from the patient and family members. Tissue used in this study was derived from drug-refractive epilepsy patients and  from regions resected from patients with deep tumours (Supplementary Tables 1, 2).

The post-mortem human brain tissue used in this study was obtained from the Neurological Foundation of New Zealand Human Brain Bank in the Centre for Brain Research at the University of Auckland. The human brain tissue was donated with informed consent from the families of the donors and its use was approved by the University of Auckland Human Participants Ethics Committee (Ref: 011654). All experiments were performed in accordance with relevant guidelines and regulations. The normal cases used in this study had no clinical history of neurological disease and no apparent pathological abnormalities upon post-mortem examination. Pathological examination by a neuropathologist confirmed the clinical diagnosis of PD by observed presence of Lewy bodies in the substantia nigra as well as pigment incontinence and cell loss in the substantia nigra. No other neuropathological changes or clinical history of other neurodegenerative diseases were present (Supplementary Tables 3, 4).

### Isolation of primary human brain pericytes

Tissue from biopsy human brains were collected as previously described^20,71^. Briefly, the tissue was mechanically dissected and dissociated before enzymatic digestion in Hank’s balanced salt solution (HBSS), containing 2.5 U/mL papain (Worthington) and 100 U/mL DNase 1 (Invitrogen) for 30 min at 37 °C with gentle rotation. This step included a gentle titration at 15 min. Following this, complete media, DMEM:F12 (Invitrogen) was used to stop the enzymatic digestion. To collect the cells, they were centrifuged (170×*g*, 10 min) and resuspended in complete media. Cells were plated onto uncoated T75 culture flasks (Nunc). For all the experiments presented here, pericytes were passage 5–9 to ensure no contamination of astrocytes or microglia in our pericyte pure cultures. Late passages (> 4) displayed immunocytochemical staining for pericyte markers such as platelet-derived growth factor receptor beta (PDGFRβ), alpha smooth muscle actin (αSMA) and neural/glial antigen 2 (NG2)^21^. The cultures also displayed immunocytochemical staining for fibroblast markers prolyl-4-hydroxylase (P4H) and fibronectin. Cells were subsequently incubated at 37 °C with 5% carbon dioxide until seeded for experiments, grown in DMEM:F12 (Invitrogen) containing 10% fetal bovine serum (Gibco) and 1% Penicillin/Streptomycin (Gibco).

### Isolation of primary human brain microglia

Microglial isolation was performed as described previously^20^. Following collection, biopsy tissue samples were thoroughly washed with Hibernate A solution (Gibco). The tissue was mechanically dissected and dissociated before enzymatic digestion in media containing DNase (10 U/mL, Invitrogen) and papain (2.5U/mL Worthington) in Hibernate A (Gibco) and incubated for 15 min at 37 °C while undergoing continuous rotation. Enzyme mixture containing tissue was triturated 10 times, before being re-incubated at 37 °C undergoing gentle rotation. Another 10 triturations were performed, and the media containing tissue was passed through a 70 µm cell strainer (Falcon) into 20 mL of no growth factor stem cell media comprised of Dulbecco’s Modified Eagle Medium: Nutrient Mixture F-12 (DMEM:F12; Gibco), supplemented with B27 (2%; Invitrogen), 1% penicillin–streptomycin (Gibco) and 1% GlutaMAX (GMAX; Invitrogen) and centrifuged at 160×*g* for 5 min. The cells were re-suspended in an appropriate amount of growth factor stem cell media supplemented with 40 ng/mL of recombinant human EGF (Peprotech), 40 ng/mL FGF-2 (Peprotech) and 2 µg/mL heparin (Sigma). Cells were then seeded in a T25 (25 cm^2^) or T75 (75 cm^2^) culture flask (Nunc) for cell culturing at 37 °C with 5% CO_2_. The following day, the cell culture flask was tapped and washed with 10 mL of DMEM:F12 to remove any non-microglial cells. This wash step was repeated 4–5 times until a pure microglial population was achieved. Cell adherence was observed under a microscope after each wash step. Microglia were subsequently maintained in DMEM:F12 (Gibco) at 37 °C with 5% CO_2_ and maintained for 4–7 days before plating.

### Cell plating

Cells were harvested for experiments by adding 2.5 mL 0.25% Trypsin-1 mM ethylenediaminetetraacetic acid (EDTA; Gibco) and incubated for 2–5 min at 37 °C to allow for cell detachment. A sterile scraper was then gently passed across the plate to remove any adherent cells. Cells were then collected in warm DMEM:F12 media. 10 µL of 1:1 Trypan Blue (Gibco) and cell suspension was prepared and added to a hemocytometer to allow for cell counting. Cells were re-suspended in the correct volume of DMEM:F12 to achieve a cell density of 5000 cells/well in a 96-well plate (Nunc). All plates were incubated for 3 days at 37 °C with 5% CO_2_ to allow for cell adherence before treatments were added.

### α-Syn expression, purification and assembly into two structurally distinct fibrillar polymorphs

Human wild type α-syn was expressed in *E. coli* BL21 DE3 CodonPlus cells (Stratagene, San Diego, CA, USA) and purified and assembled into the fibrillar polymorphs as described previously^72^. Briefly, the protein (200 µM) was incubated in 50 mM Tris–HCl, pH 7.5, 150 mM KCl for the polymorph fibrils in 5 mM Tris pH 7.5 for the polymorph “ribbons” at 37 °C under continuous shaking in an Eppendorf Thermomixer set at 600 rpm for 10 days while withdrawing aliquots (5 µL) at different time intervals, mixing them to Thioflavin T (10 µM final) and recording the fluorescence increase on a Cary Eclipse Fluorescence Spectrophotometer (Varian Medical Systems Inc., Palo Alto, CA, USA) using an excitation wavelength = 440 nm, an emission wavelength = 480 nm and excitation and emission slits set at 2 and 5 nm, respectively. Assembly reaction completion was further assessed by sedimentation at 100,000 × *g* at 25 °C for 30 min and measurement of the amount of protein remaining in the supernatant. The fibrillar nature of the resulting aggregates was assessed by transmission electron microscopy after negative staining.

### Cytokine and α-synuclein treatments

Inflammatory cytokines interleukin-1β (IL-1β) (Peprotech, NJ, USA), and lipopolysaccharide (LPS) (from *Escherichia coli* 026:B6, L4391, Sigma, MO, USA) or vehicle (PBS or 0.1% BSA in PBS) were added to pericytes (Table 1). Two types of fibrillar polymorphs with features that are similar to those seen in two synucleinopathies—ribbons and fibrils^69,72–76^ or vehicle (PBS or 0.1% BSA in PBS) were added to pericytes (Table 1).

Fibrils and ribbons were fragmented by sonication for 5 min in 2 mL Eppendorf tubes in a Vial Tweeter powered by an ultrasonic processor UIS250v (200 W, 2.4 kHz; Hielscher Ultrasonic, Teltow, Germany), aliquoted, flash-frozen in liquid nitrogen and stored at − 80 °C until they were added to the cells. Electron micrographs of fibrils and ribbons are shown in Supplementary Fig. 14.

### Electron microscopy

α-Syn fibrillar polymorphs were visualized before and after fragmentation by electron microscopy after negative staining with uranyl acetate (Supplementary Fig. 14). Firstly, α-syn fibrillar polymorphs were diluted to a final concentration of 1 μM. 10 µL of diluted α-syn was deposited onto a 200 mesh copper carbon coated grids (Sigma MO, USA) for 2 min. The copper grid was then floated on 50 µL drop of 1% Uranyl acetate pH 8.0 for 1 min and then blotted again. α-syn fibrils absorbed onto the copper grid were imaged using a Jeol 1400 transmission electron microscope. The images were recorded using a Gatan Orius CCD camera (Gatan Inc., Pleasanton, CA, USA).

Transmission electron microscopy (TEM) reveals that the two α-syn polymorphs have different physical characteristics. Fibrils (Supplementary Fig. 14a) are more cylindrical while ribbons (Supplementary Fig. 14b) are flat^72^.

### Thioflavin T assay

In a clean microcentrifuge tube, 5 μL of either ribbons or fibrils were added to 245 μL of 1× PBS to dilute the α-syn 1:50. Next, 250 μL of 1× PBS was added to separate microcentrifuge tube to act as a negative control. Next, 250 μL of 25 μM thioflavin T (Sigma) was added to each sample and gently mixed. Two replicates, 200 μL each were placed into a 96 well plate (Nunc) and kept in the dark to prevent photobleaching. The α-syn was incubated for 1 h at room temperature with the thioflavin T and the plate was read at excitation of 450 nm and emission of 510 nm. Fibrils displayed an increase in Thioflavin T (ThT) binding after 1 h of incubation when compared to the negative control, whereas ribbons did not^72^ (Supplementary Fig. 15).

### Immunocytochemistry

Cells were fixed using 4% paraformaldehyde (PFA) for 15 min at room temperature and washed in phosphate buffered saline with 0.1% Triton X-100 (PBS-T). Cells were incubated with primary antibodies—nuclear Factor kappa-light-chain-enhancer of activated B cells (NF-κB) p65 (Abcam, ab32536, 1:500), PDGFRβ (Y92) (Abcam, ab32570, 1:500), intracellular adhesion molecule-1 (ICAM-1) (Santa Cruz, sc-107, 1:500), monocyte chemoattractant protein-1 (MCP-1) (Abcam, ab9669, 1:500), α-syn (Abcam, ab1903, 1:3000), cleaved caspase-3 (Abcam, ab2302, 1:500) and LAMP-1 (DHSB H4A3, 1:500) overnight at 4 °C, diluted in PBS containing 1% normal goat serum. Cells were washed in PBS-T and incubated with appropriate anti-species fluorescently conjugated secondary antibodies for three hours at room temperature. The cells were washed again with PBS-T and incubated with Hoechst 33342 (1:500, Molecular probes # H1399) for 15 min at room temperature to counterstain cell nuclei. Images were acquired using the automated fluorescence microscope ImageXpress Micro XLS (Version 5.3.0.1, Molecular Devices, CA, USA) using the 20× (0.45 NA) CFI Super Plan Fluor ELWD ADM objective lens and Lumencor Spectra X configurable light engine source. Quantitative analysis of several measures including percentage positive cytoplasmic staining, cytoplasmic intensity measures and nuclear transcription factor location were performed using the cell scoring and integrated morphometry analysis modules on MetaXpress software (Version 5.3.0.1, Molecular Devices, CA, USA). Roughly 500–1000 cells were scored per well with multiple wells (at least three) analysed per sample.

### Cytometric bead array analysis

Conditioned media samples from pericytes and microglia treated as above were spun at 200×*g* for 5 min and the supernatant collected and stored at − 20 °C. The concentration of cytokines was measured using a cytometric bead array (CBA) (BD Biosciences, CA, USA) as per manufacturer’s instructions. CBA samples were run on an Acuri C6 flow cytometer (BD Biosciences, CA, USA). Data were analysed using FCAP-array software (version 3.1; BD Biosciences, CA, USA) to convert fluorescent intensity values to concentrations using an 11-point standard curve (0–10,000 pg/mL) (Supplementary Table 4).

### Secretome profiler analysis

Conditioned media samples from pericytes and microglia treated as above for 24 h were spun 200×*g* for 5 min and the supernatant collected and stored at − 20 °C. The conditioned media was assayed using the Proteome Profiler Human XL Cytokine Array Kit as per manufacturer’s instructions (R&D Systems, MN, USA). Briefly, membranes spotted with antibodies were incubated with conditioned media overnight at 4 °C. The following day, a detection antibody cocktail was added for 1 h at room temperature, before visualization using chemiluminescence. Images were acquired using the Li-COR Odyssey FC imaging system, and spot intensity was quantified using ImageJ and normalized to reference spots.

### Toll-like receptors

Ribbons, fibrils or vehicle (PBS or 0.1% BSA in PBS) (Table 1) were added to pericytes for 24 h, after which TLR ligands (Supplementary Table 6) were added. After 24 h of TLR ligand treatment, conditioned media was taken and analysed by CBA as detailed above. Immunocytochemistry for NF-κB p65 (Abcam, ab32536, 1:500) was performed 1 h after treatment with the TLR ligands.

### Endotoxin quantification

The quantification of endotoxins in the of α-syn aggregates, vehicle, LPS and IL-1β was carried out using the Pierce Chromogenic Endotoxin Quant Kit (A39552, Thermofisher) as per the manufacturer’s instructions (Table 2). Briefly, the endotoxin standards, samples and blanks were all run in triplicate. To run the standards and samples, a 96 well plate was pre-equilibrated by being slightly submerged in a 37 °C water bath and remained there for the remainder of the assay. Endotoxin standard, blanks and α-syn preparations (100 nM) were added to each well followed by the addition of reconstituted Amebocyte Lysate Reagent and incubated for 60 min. Next, Reconstituted Chromogenic Substrate Solution was added to each well and incubated for 6 min. After 6 min, Stop Solution (25% acetic acid) was added. Optical density (OD) at 405 nm was read immediately after assay completion using a Synergy 2 Multi-Mode Microplate Reader (BioTek, VT, USA). Concentrations of endotoxins were calculated using a standard curve with a coefficient of determination, r^2^, ≥ 0.98.

**Table 2:** Quantification of endotoxins in the α-syn preparations and inflammatory stimuli used on primary human brain pericytes and microglia.

Treatment	Endotoxin units/mL (EU/mL)
Vehicle	0
Ribbons	0
Fibrils	0
LPS	4.81
IL-1β	0

### Cell viability assay

To measure cell viability after cytokine and α-syn treatment, ReadyProbes Cell Viability Imaging Kit (Molecular Probes) was used. Two drops of both NucBlue Live Reagent and NucGreen Dead reagent was added to 1 mL of DMEM/F12, 10% FBS and 1% PSG and a complete media change was performed on the cells. After a 15 min incubation at 37 °C, live imaging of healthy or compromised cells were acquired using the automated fluorescence microscope ImageXpress Micro XLS (Version 5.3.0.1, Molecular Devices, CA, USA) using the 20× (0.45 NA) CFI Super Plan Fluor ELWD ADM objective lens and Lumencor Spectra X configurable light engine source.

### Fixed flow cytometry

Pericytes were cultured in 24 well plates (Nunc) before treatment with α-syn or vehicle (PBS). Following timed treatments, cultures were incubated with Trypsin-1 mM EDTA (Gibco) at 37 °C for 5 min to harvest cells and placed in FACS tubes (Falcon). Cells in suspension were incubated in 8% PFA for 10 min and then centrifuged at 300×*g* for 5 min. Subsequently cells were washed twice with PBS-T for permeabilization for 10 min each. Cells were incubated with primary antibodies at 1:500, including a no-primary control overnight at 4 °C. The cells were pelleted, and the primary antibody removed and cells were washed before addition of secondary antibody including the nuclear stain 7-Amino-Actinomcyin D (7-AAD) (BD Pharmingen; 559925, 1:100) for 3 h at room temperature. Samples were then run on Accuri C6 flow cytometer (BD Biosciences) until 5000 cellular events had been recorded (Supplementary Fig. 10).

### Degradation inhibitors

To inhibit the two degradation pathways in pericytes they were treated with either a proteasome inhibitor MG132 (Abcam, ab141003, 5 µM) for 6–8 h or a lysosomal inhibitor bafilomycin A1, (V)-ATPase inhibitor (Abcam, ab120497, 200 nM) for 4–6 h or vehicle (DMSO).

### Lysosomal activity assay

To assess the functionality of the lysosomal system in pericytes a lysosomal intracellular activity assay kit (Abcam, ab234622) was used. Briefly, the media was removed and replaced with fresh complete medium containing vehicle, positive control (Bafilomycin) and the test compound at desired concentrations. The cells were incubated for 1 h at 37 °C with 5% CO_2_. Next, the media was removed and replaced with complete fresh medium. Next, 15 µL of Self-Quenched substrate per 1 mL of media was added to each experimental and control well. Cells were subsequently incubated for 1 h at 37 °C with 5% CO_2_. To stop the reaction, the cells were washed twice with 1 mL ice cold Assay Buffer and the cells were imaged under a fluorescent microscope with 488 nm excitation filter. Images were acquired using the automated fluorescence microscope ImageXpress Micro XLS (Version 5.3.0.1, Molecular Devices, CA, USA) using the 20× (0.45 NA) CFI Super Plan Fluor ELWD ADM objective lens and Lumencor Spectra X configurable light engine source. Quantitative analysis of several measures including percentage positive cytoplasmic staining, cytoplasmic intensity measures and granularity measurements were performed using the cell scoring, granularity and integrated morphometry analysis modules on MetaXpress software (Version 5.3.0.1, Molecular Devices, CA, USA). Roughly 500–1000 cells were scored per well with multiple wells (at least three) analysed per sample.

### CellROX

Pericytes were either pre-treated with MG132 (5 µM) for 6 h prior to incubation with α-syn ribbons or fibrils for 24 h. CellROX reagent was added to the cells at a final concentration of 5 µM. The cells were subsequently incubated for 30 min at 37 °C. 5% CO_2_. To label cell nuclei, the NucBlue Live Cell counterstain was added at the same time as the CellROX reagent. The cells were subsequently imaged using the automated fluorescence microscope ImageXpress Micro XLS (Version 5.3.0.1, Molecular Devices, CA, USA) using the 20× (0.45 NA) CFI Super Plan Fluor ELWD ADM objective lens and Lumencor Spectra X configurable light engine source. Quantitative analysis of several measures including percentage positive cytoplasmic staining, cytoplasmic intensity measures and granularity measurements were performed using the cell scoring, granularity and integrated morphometry analysis modules on MetaXpress software (Version 5.3.0.1, Molecular Devices, CA, USA). Roughly 500–1000 cells were scored per well with multiple wells (at least three) analysed per sample.

### MitoSOX

Pericytes were either pre-treated with MG132 (5 µM) for 6 h prior to incubation with α-syn ribbons or fibrils for 24 h. MitoSOX reagent was added to the cells at a final concentration of 5 µM. The cells were subsequently incubated for 30 min at 37 °C. 5% CO_2_. To label cell nuclei, the NucBlue Live Cell counterstain was added at the same time as the CellROX reagent. The cells were subsequently imaged using the automated fluorescence microscope ImageXpress Micro XLS (Version 5.3.0.1, Molecular Devices, CA, USA) using the 20 x (0.45 NA) CFI Super Plan Fluor ELWD ADM objective lens and Lumencor Spectra X configurable light engine source. Quantitative analysis of several measures including percentage positive cytoplasmic staining, cytoplasmic intensity measures and granularity measurements were performed using the cell scoring, granularity and integrated morphometry analysis modules on MetaXpress software (Version 5.3.0.1, Molecular Devices, CA, USA). Roughly 500–1000 cells were scored per well with multiple wells (at least three) analysed per sample.

### Statistical analysis

All experiments were performed in at least three independent cases. In general, data are presented as mean ± standard deviation (SD) from at least three independent experiments. Data visualization and statistical hypothesis testing was performed using GraphPad Prism Version 8.02, Python 3.9.9 and the Seaborn 0.11.2 library. Two-way analysis of variance (ANOVA) was used when comparing time across the cytokine and inflammatory stimuli treatments with Tukey’s multiple comparisons adjustment. One-way ANOVA with Tukey’s multiple comparison adjustment was used when comparing responses between treatments. Statistical significance was set as p < 0.05.

### Ethics approval and consent to participate

The use of human tissue and collection procedures were approved by the Northern Regional Ethics Committee (New Zealand) for biopsy tissue (AKL/88/025). Biopsy human brain tissue was obtained with informed written consent from the patient and family members. The post-mortem human brain tissue used in this study was obtained from the Neurological Foundation of New Zealand Human Brain Bank in the Centre for Brain Research at the University of Auckland. The human brain tissue was donated with consent from the families of the donors and its use was approved by the University of Auckland Human Participants Ethics Committee (Ref: 011654).

## Supplementary Information

Supplementary Information.

## Data Availability

The datasets generated during and/or analysed during the current study are available from the corresponding author on reasonable request.
