# Supplementary material for: Pericytes take up and degrade α-synuclein but succumb to apoptosis under cellular stress
Source: Sci Rep. 2022 Oct 15;12:17314. doi: 10.1038/s41598-022-20261-0 (PMC9569325; doi:10.1038/s41598-022-20261-0)
Supplement: Supplementary file 1 — Supplementary Information. [file 41598_2022_20261_MOESM1_ESM.docx]

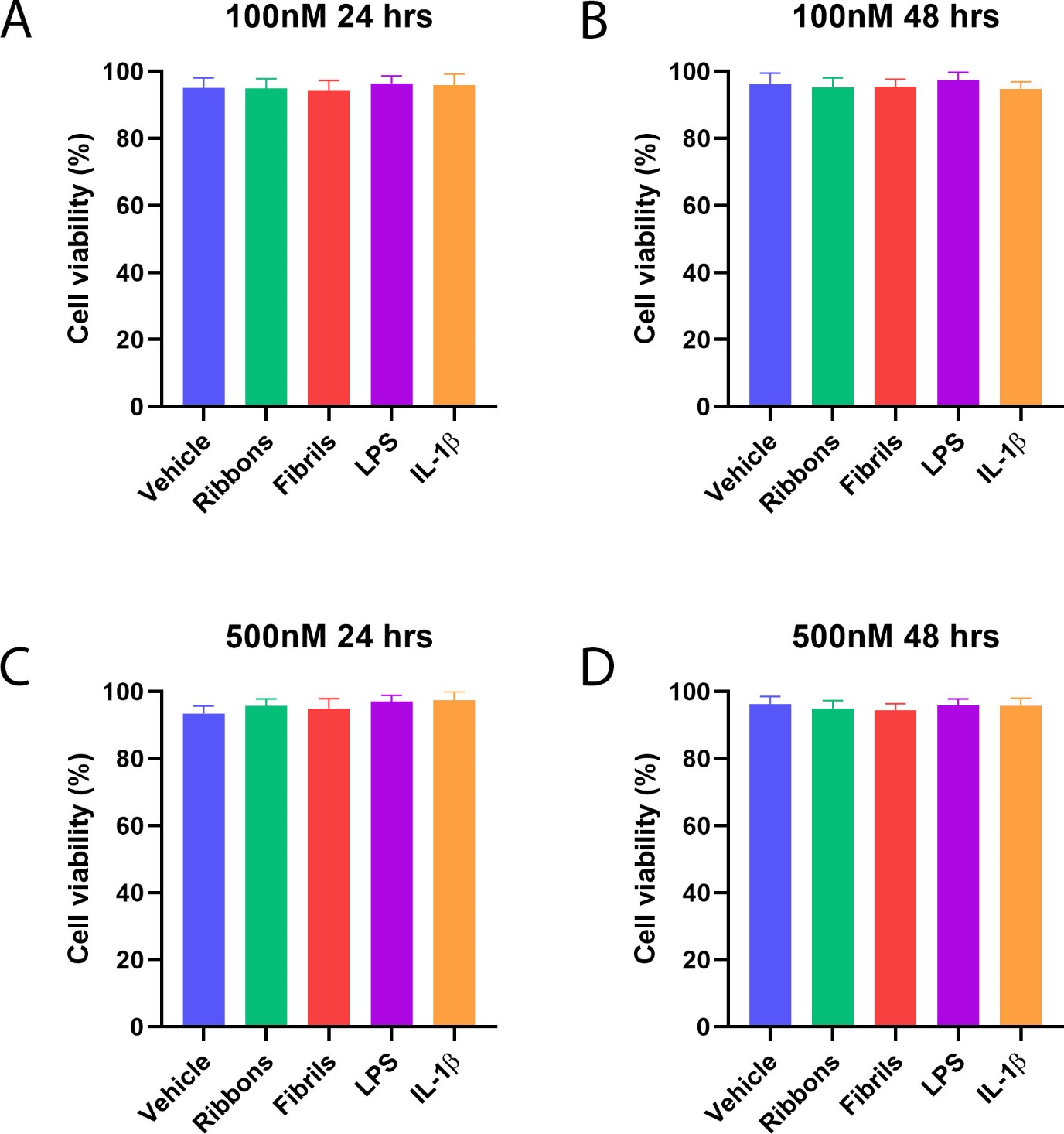


Supplementary figure 1: Cell viability of pericytes treated with α-syn and inflammatory stimuli

Measurement of cell viability in pericytes after treatment with α-syn at 100 nM for (A) 24 hours, (B) 48 hours and at 500 nM for (C) 24 hours and (D) 48 hours. LPS and Il-1β were treated at 10 ng/mL (n = 3, mean ± SD).


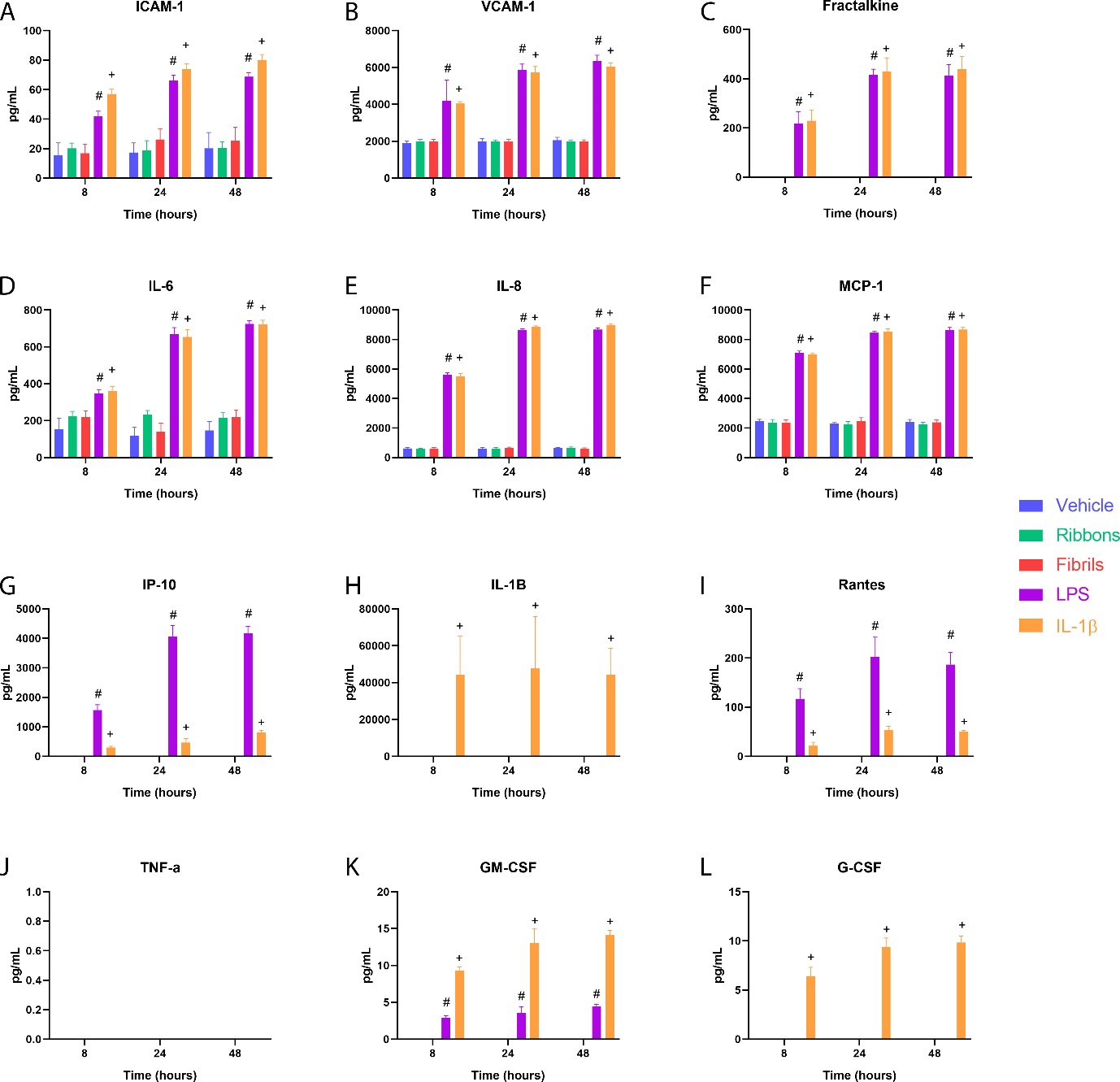


Supplementary figure 2: Inflammatory responses of pericytes measured by CBA

Pericytes were cultured and treated with vehicle, two different α-syn preparations (100 nM), LPS (10 ng/mL) and IL-1β (10 ng/mL) before conditioned media were taken and cytokine secretion analysed by cytometric bead array. Secretion of (A) ICAM-1, (B) VCAM-1, (C) Fractalkine, (D) IL-6, (E) IL-8, (F) MCP-1, (G) IP-10, (H) IL-1β, (I) RANTES, (J) TNF-α, (K) GM-CSF and (L) G-CSF at 8h, 24 and 48h after treatment. (n = 3, mean ± SD). *# LPS, + IL-1β vs vehicle p < 0.05*


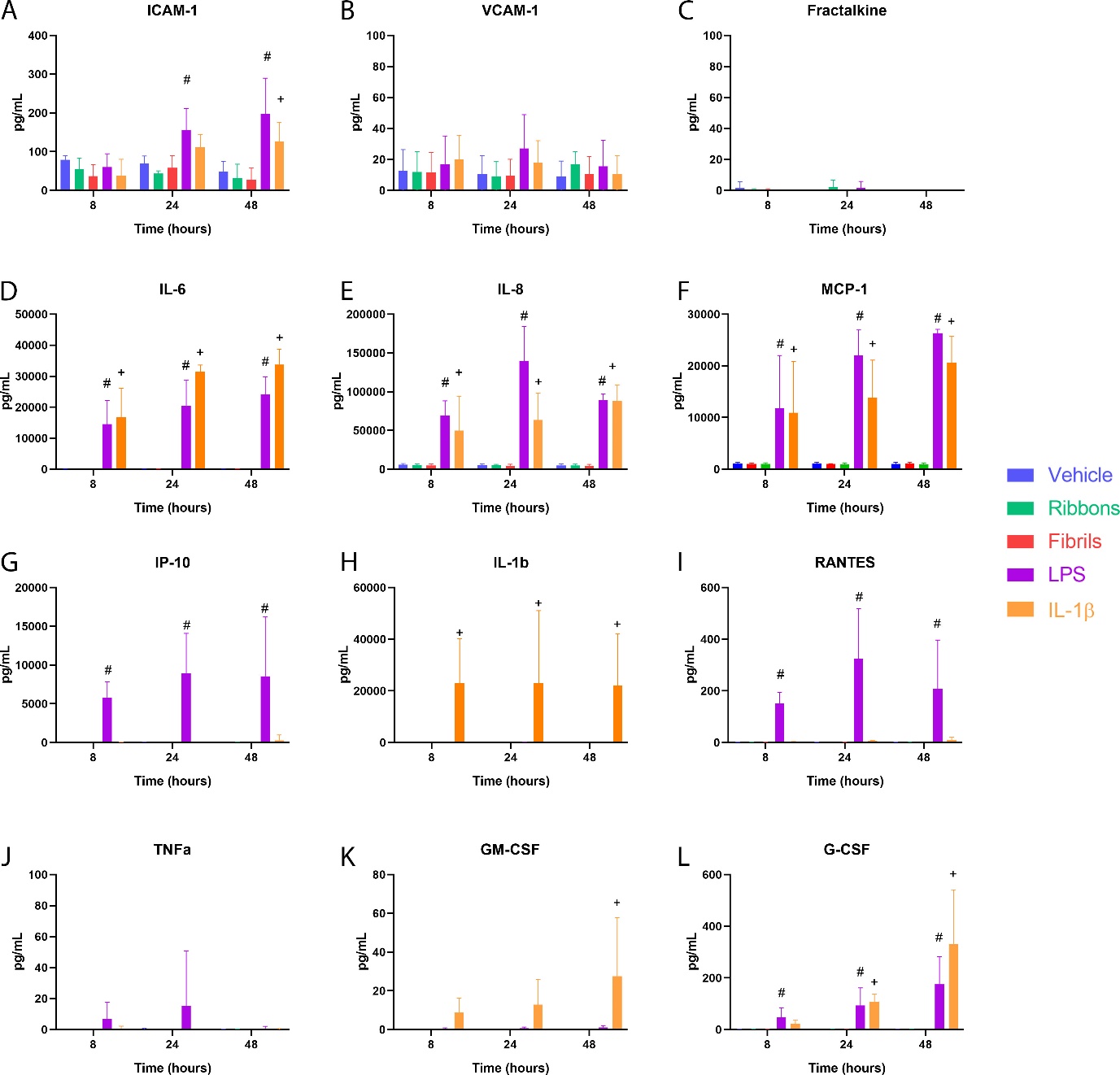


Supplementary figure 3: Inflammatory responses of microglia measured by CBA

Microglia were cultured and treated with vehicle, two different α-synuclein preparations (100 nM), LPS (10 ng/mL) and IL-1β (10 ng/mL) before conditioned media were taken and cytokine secretion analysed by cytometric bead array. Secretions of (A) ICAM-1, (B) VCAM-1, (C) Fractalkine, (D) IL-6, (E) IL-8, (F) MCP-1, (G) IP-10, (H) IL-1β, (I) RANTES, (J) TNF-α, (K) GM-CSF and (L) G-CSF at 8h, 24 and 48h after treatment. (n = 3, mean ± SD). *# LPS, + IL-1β vs vehicle p < 0.05*


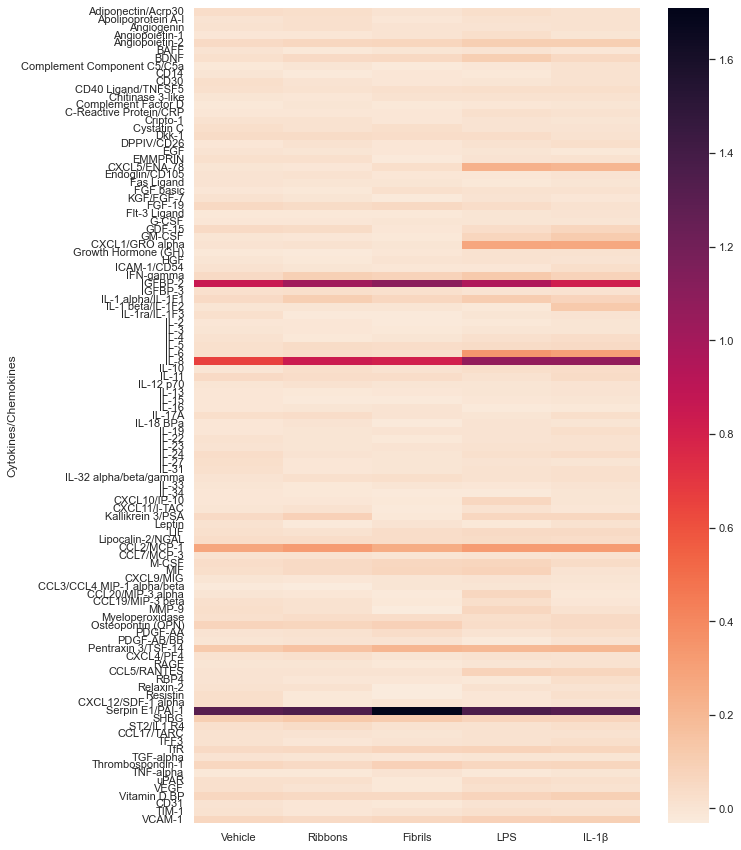


Supplementary Figure 4: Inflammatory responses of pericytes measured by a proteome profiler

Pericytes were cultured and treated with vehicle, two different α-synuclein preparations (100 nM), LPS (10 ng/mL) and IL-1β (10 ng/mL) for 24h before conditioned media were taken and cytokine secretion measured using a Proteome Profiler™ Human XL Cytokine Array Kit. Heatmap analysis of secretions in pericytes (n = 1).


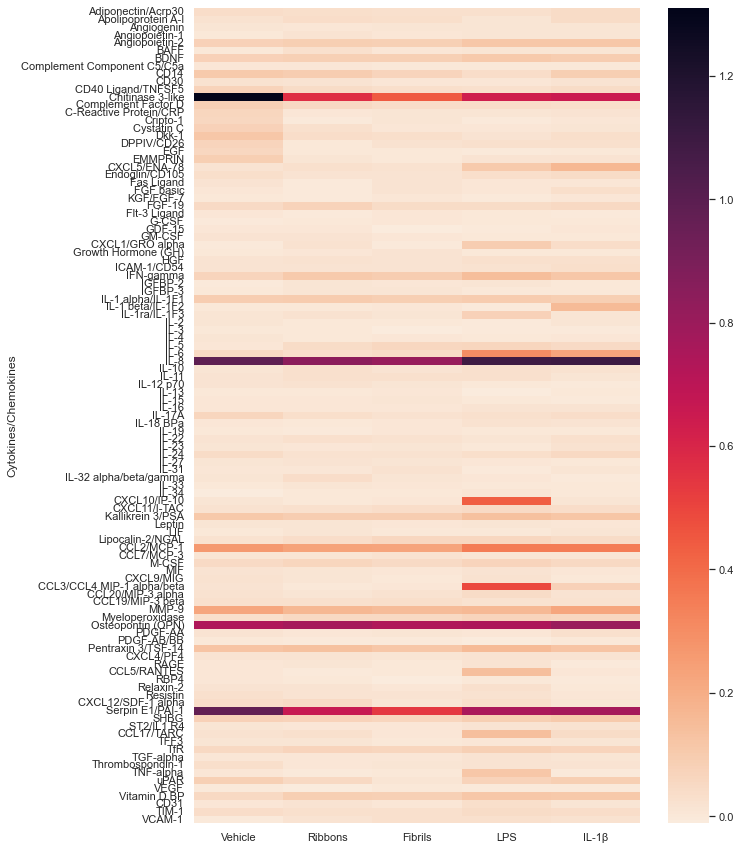


Supplementary Figure 5: Inflammatory responses of microglia measured by a proteome profiler

Microglia were cultured and treated with vehicle, two different α-synuclein preparations (100 nM), LPS (10 ng/mL) and IL-1β (10 ng/mL) for 24h before conditioned media were taken and cytokine secretion measured using a Proteome Profiler™ Human XL Cytokine Array Kit. Heatmap analysis of secretions in microglia (n = 1).


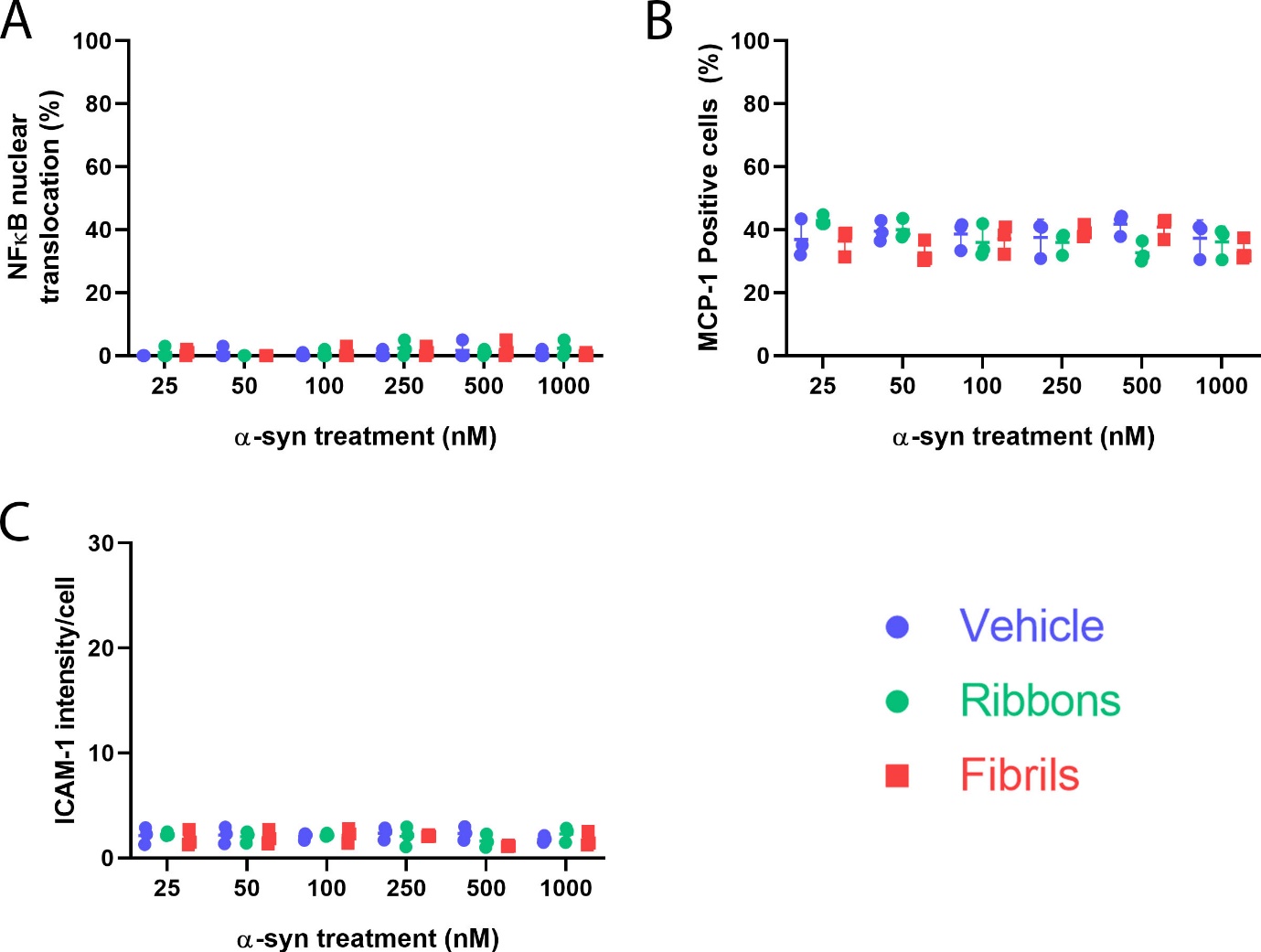


Supplementary figure 6: Pericytes response to increasing concentrations of α-syn

Immunocytochemistry quantification of pericytes when treated with either vehicle, ribbons (25 – 1000 nM) and fibrils (25 – 1000 nM). (A) NF-κB nuclear translocation after 1h treatment (B) MCP-1 and (C) ICAM-1 induction after 24h (n = 3, mean ± SD). *p > 0.05* when compared to each concentration by Two-way ANOVA with Tukey’s multiple comparisons test.


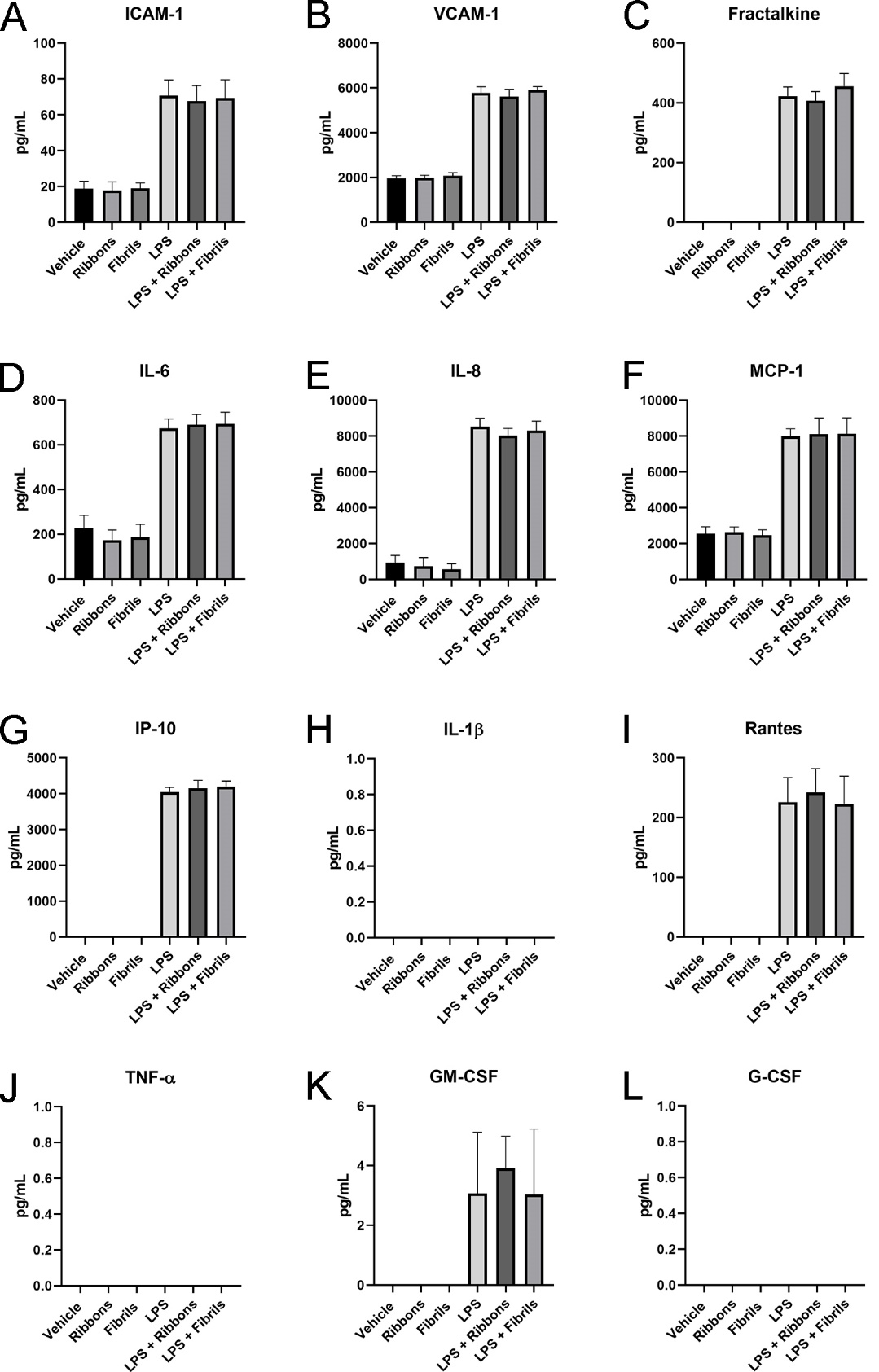


Supplementary figure 7: Inflammatory responses of pericytes when co-treated with LPS and α-syn

Pericytes were cultured and treated with vehicle, ribbons (100 nM), fibrils (100 nM), LPS (10 ng/mL) and LPS co-treated with ribbons or fibrils before conditioned media was taken and cytokine secretions analysed by cytometric bead array. Secretions of (A) ICAM-1, (B) VCAM-1, (C) Fractalkine, (D) IL-6, (E) IL-8, (F) MCP-1, (G) IP-10, (H) IL-1β, (I) RANTES, (J) TNF-α, (K) GM-CSF and (L) G-CSF at 24h after treatment (n = 3, mean ± SD). *p > 0.05* when compared to LPS treatment by One-way ANOVA with Tukey’s multiple comparisons test.


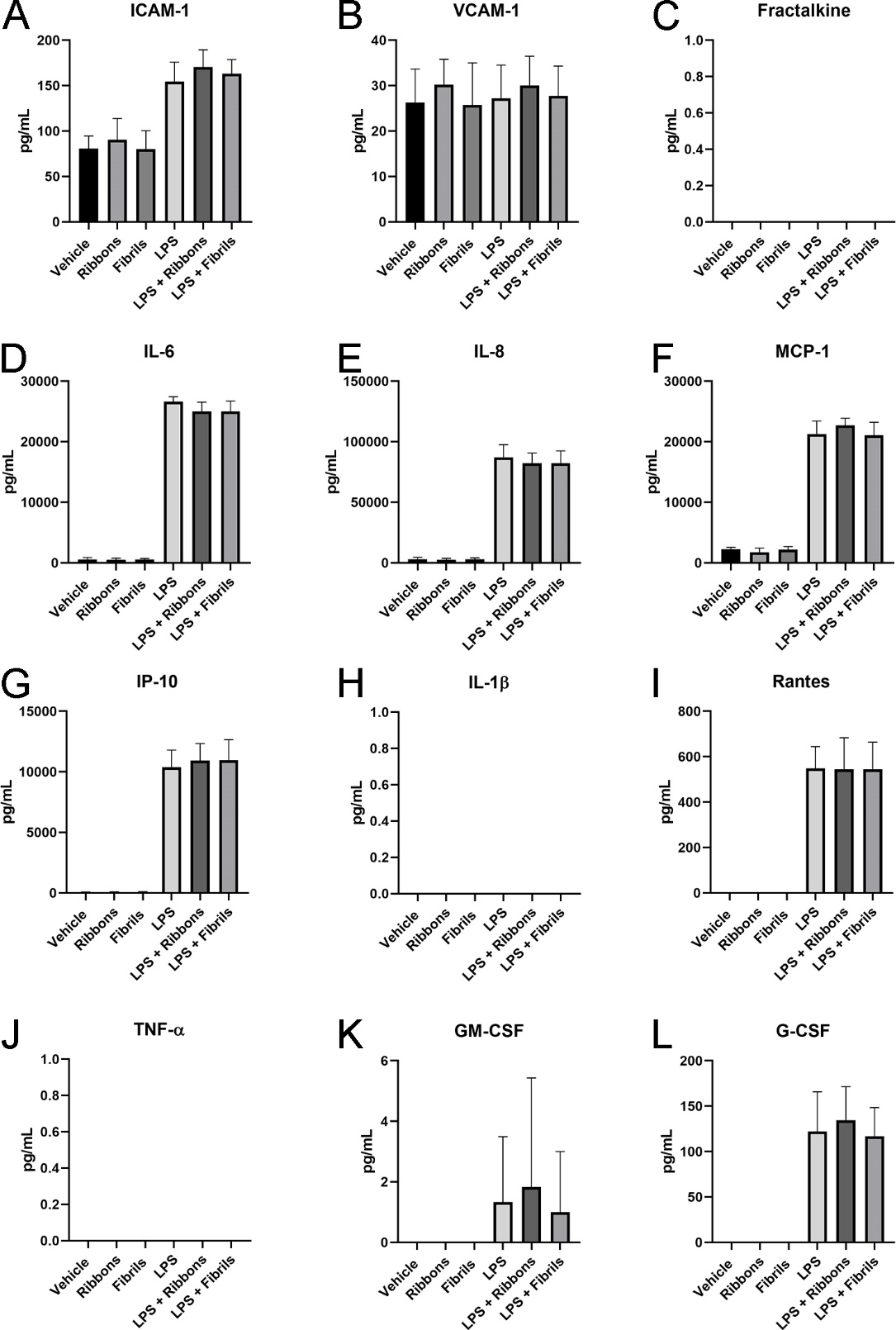


Supplementary figure 8: Inflammatory responses of microglia when co-treated with LPS and α-syn

Microglia were cultured and treated with vehicle, ribbons (100 nM), fibrils (100 nM), LPS (10 ng/mL) and LPS co-treated with ribbons or fibrils before conditioned media was taken and cytokine secretions analysed by cytometric bead array. Secretions of (A) ICAM-1, (B) VCAM-1, (C) Fractalkine, (D) IL-6, (E) IL-8, (F) MCP-1, (G) IP-10, (H) IL-1β, (I) RANTES, (J) TNF-α, (K) GM-CSF and (L) G-CSF at 24h after treatment (n = 1, mean ± SD). *p > 0.05* when compared to LPS treatment by One-way ANOVA with Tukey’s multiple comparisons test.


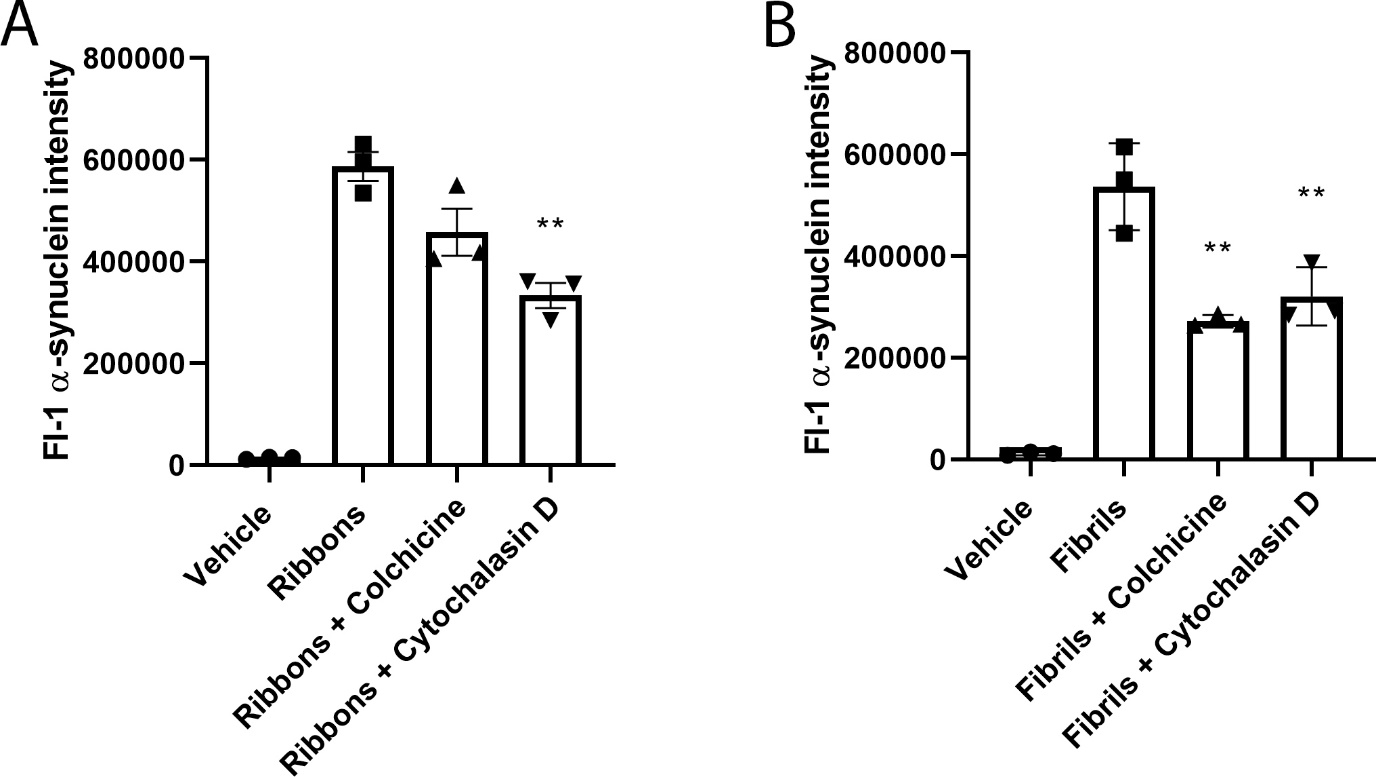


Figure 9: Inhibition of phagocytosis in primary human brain pericytes

Primary human brain epilepsy pericytes were pre-treated with cytochalasin D (20 µM), colchicine (10 µM) or vehicle (0.2% DMSO) for 30 minutes prior to a two hour incubation with ribbons (100 nM) or fibrils (100 nM). α-syn phagocytosis was measured using fixed flow cytometry to measure MFI after treatment with phagocytosis inhibitors with (A) ribbons and (B) fibrils (n = 3, mean ± SD). ** p < 0.001 when compared to the respective α-syn treatment.


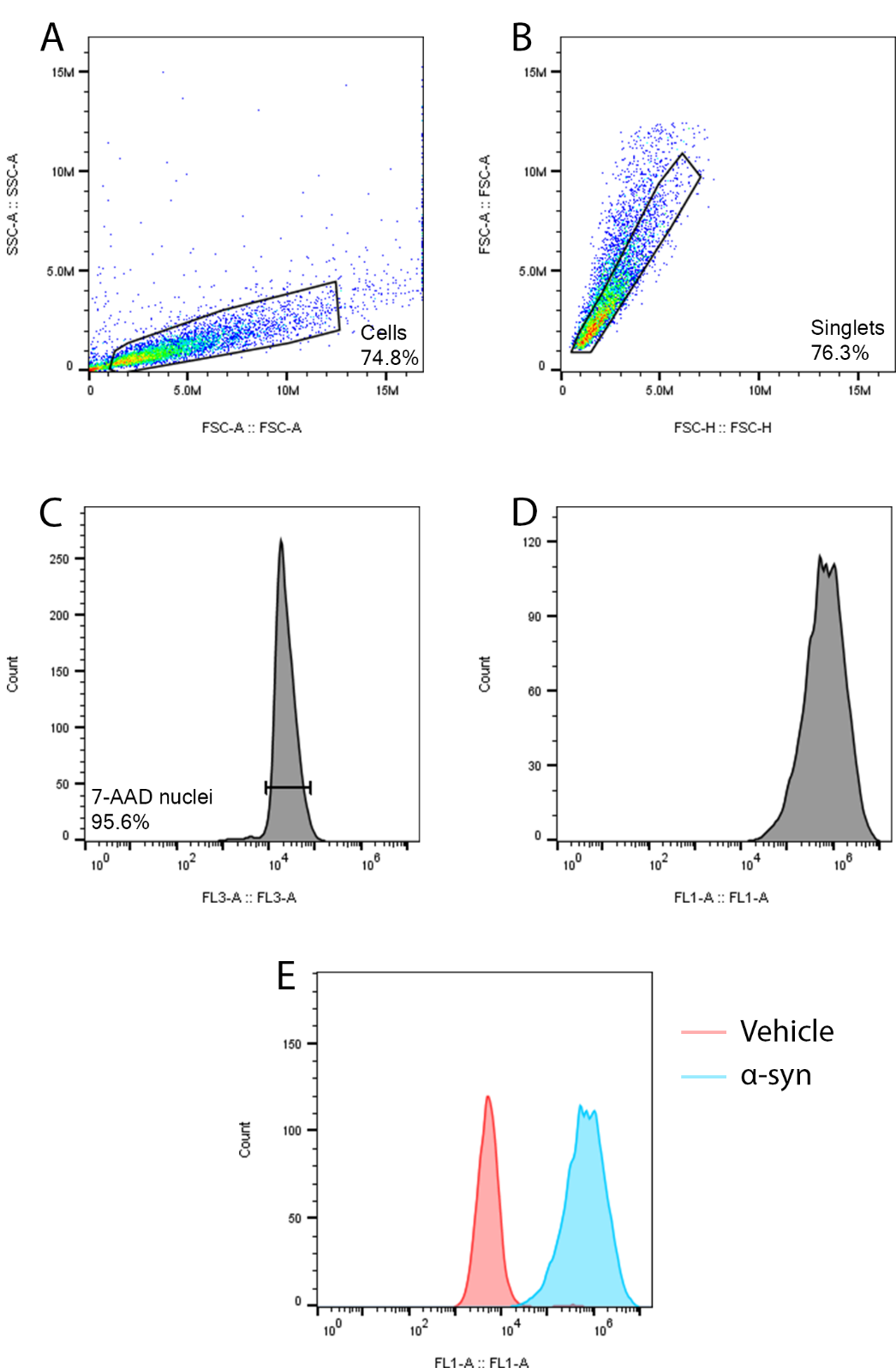


Supplementary figure 10: Methodology for measurement of intracellular α-syn using flow cytometry

Flow cytometry methodology for gate selection and quantification of the amount of intracellular α-syn in pericytes. (A) Cell selection and removal of debris using side scatter and forward scatter, (B) selection of single cells and removal of doublets using forward scatter, (C) selection of 7-AAD positive cells and lastly, (D) selection of cells that are positive for α-syn. (E) Graphic to demonstrate differences after following the procedure between vehicle and α-syn treated pericytes.


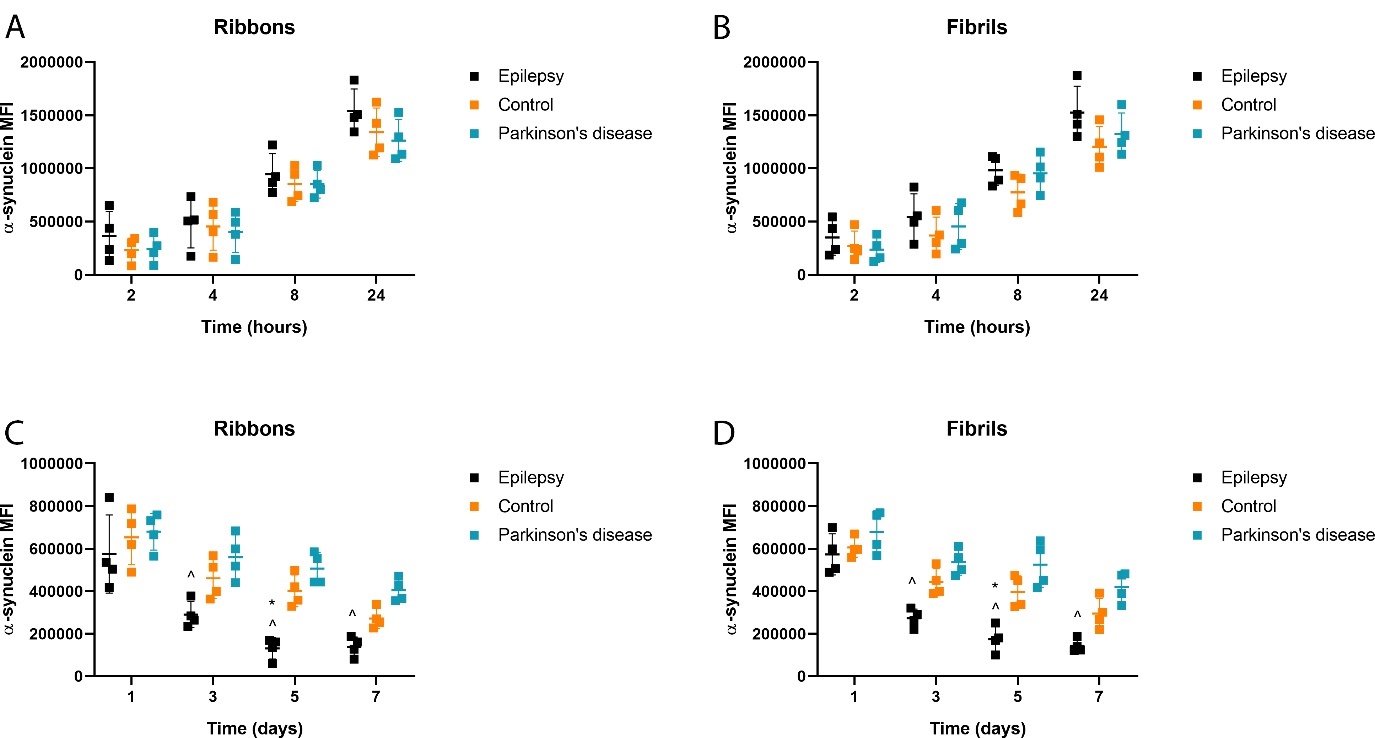


Supplementary figure 11: Comparison between ribbon and fibril α-syn aggregates and uptake of α-syn in epilepsy, control and PD pericytes over 24 hours of treatment and degradation of α-syn after 7 day incubations

Primary human brain pericytes were incubated with ribbons (100 nM) or fibrils (100 nM) over 24 hours and α-syn MFI was measured using fixed flow cytometry. Repeated measures analysis was carried out between epilepsy, control and PD pericytes for either (A) ribbons or (B) fibrils. (n = 4 in each group, mean ± SD). Primary human brain pericytes were incubated with ribbons (50 nM) or fibrils (50 nM) over 7 days and α-syn MFI was measured using fixed flow cytometry. Repeated measures analysis was carried out between epilepsy, control and PD pericytes for either (C) ribbons or (D) fibrils. *(n = 4 in each group, mean ± SD).* ** p < 0.05 compared to control pericytes ^ p < 0.05 compared to PD pericytes*


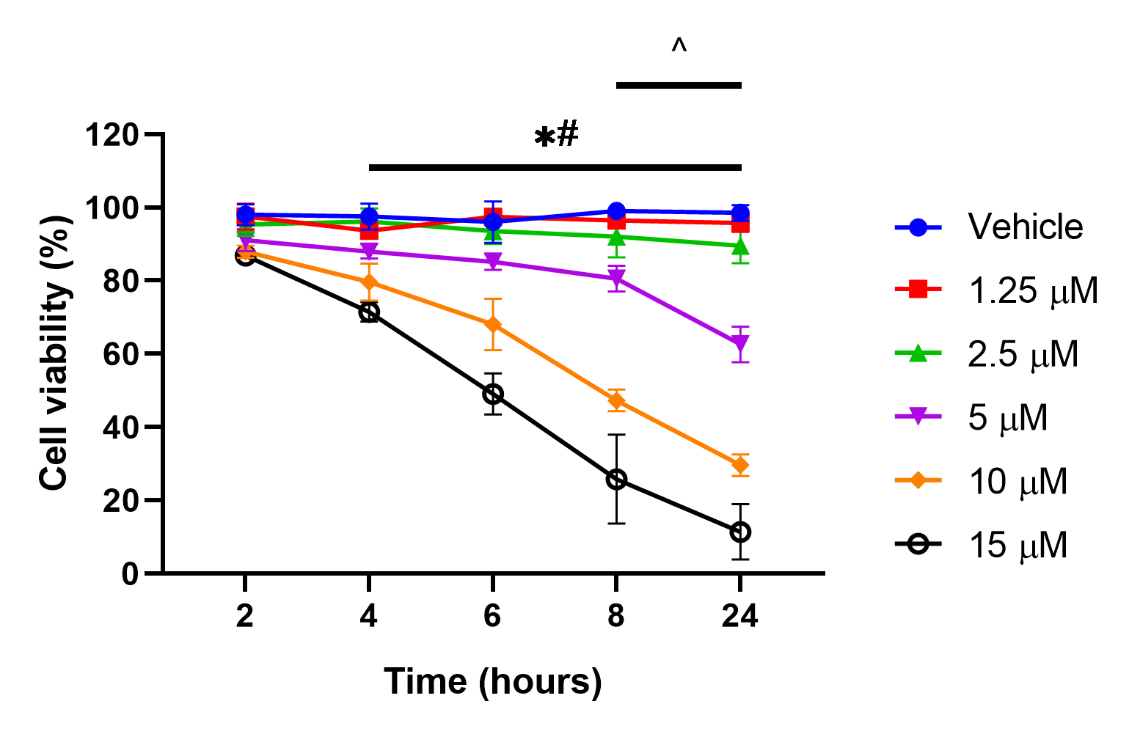


Supplementary figure 12: Primary human brain pericytes viability over 24 hours with increasing concentrations of MG132

Primary human brain pericytes were treated with MG132 (1.25 – 15 µM) or vehicle over a 24 hour incubation*. (n = 4, mean ± SD). ^ p < 0.05 5 µM compared to vehicle, * p < 0.05 10 µM compared to vehicle, # p < 0.05 15 µM compared to vehicle*


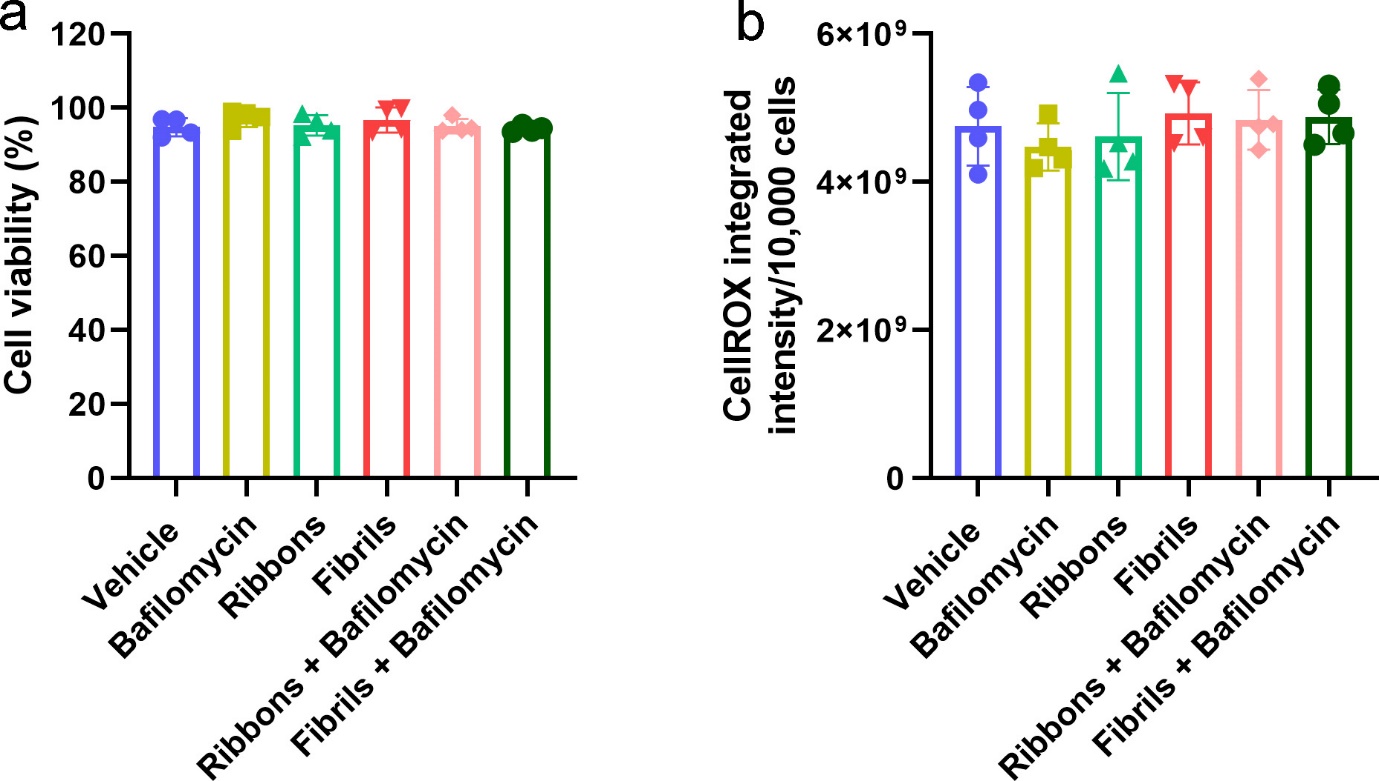


Figure 13: Cell viability and cellROX integrated intensity of primary human brain pericytes when treated with α-syn and bafilomycin

Graphs showing (A) cell viability and (B) amount of ROS production using cellROX when normalized to 10,000 cells when pericytes were pre-treated with 200 nM bafilomycin for 8 hours prior to incubation with either ribbons (100 nM) or fibrils (100 nM). (n = 4 mean ± SD).


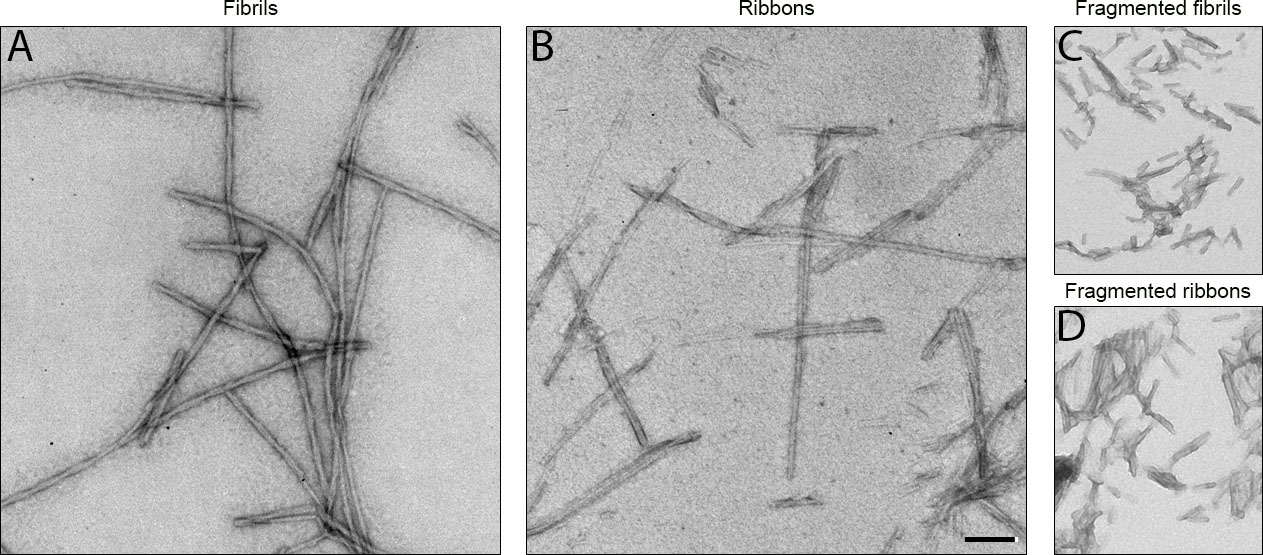


Supplementary figure 14: Negatively stained TEM of α-syn aggregates

Negative staining of α-syn aggregates using uranyl acetate before (A, B) and after fragmentation (C, D). Fragmentation by sonication for 5 min using a sonotrode (soni 0.5s pules, Sonicator UIS250V, equipped with VialTweeter, Hielscher Ultrasound Technology, Germany). Fibrils and Ribbons were imaged using a Joel 1400 transmission electron microscope following their adsorption onto carbon-coated 200 mesh grids. Scale bar, 100 nm.


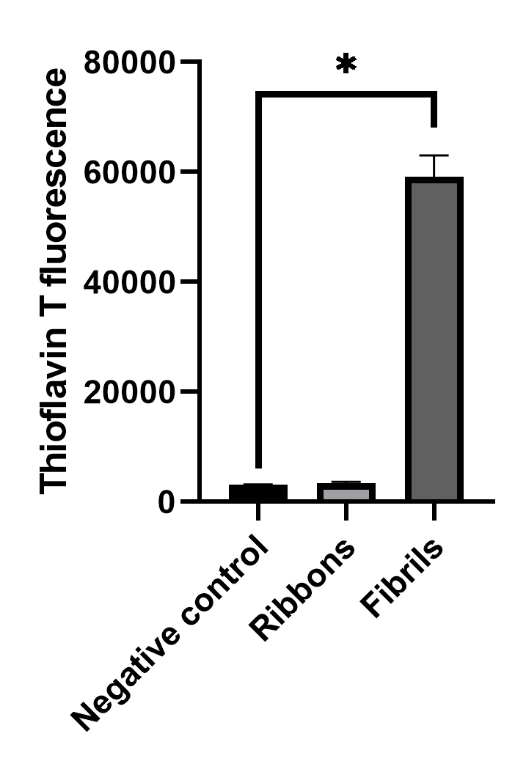


Supplementary figure 15: Thioflavin T binding to α-syn preparations

Thioflavin T binding to α-syn aggregates following a 1 hour incubation. (n = 2 ± SD) * p < 0.05 when compared to the negative control

Supplementary table 1: Case details for epilepsy tissue used to culture pericytes in this study

| **Case** | **Pathology** | **Region of resected specimen** | **Age (years)** | **Sex** |
| --- | --- | --- | --- | --- |
| E203 | Epilepsy, Mesial temporal sclerosis | Right temporal lobe | 46 | F |
| E204 | Epilepsy, hippocampal sclerosis | Right temporal lobe | 45 | F |
| E213 | Epilepsy, patchy gliosis, no cortical dysplasia, no neoplasia, presumed | Left anterior temporal lobe | 23 | M |
| E214 | Epilepsy, Mesial temporal sclerosis (Grade 4) | Left temporal lobe | 35 | F |
| E215 | Epilepsy, Mesial temporal sclerosis (Grade 3) | Right anterior and posterior temporal lobe | 29 | F |
| E216 | Epilepsy, Mesial temporal sclerosis (Grade 3) | Left temporal lobe | 38 | M |
| E217 | Epilepsy, Mesial temporal sclerosis (Grade 3) | Left anterior temporal lobe | 30 | M |

Supplementary table 2: Case details for tissue used to culture microglia in this study

| **Case** | **Pathology** | **Region of resected specimen** | **Age** | **Sex** |
| --- | --- | --- | --- | --- |
| E217 | Epilepsy, Mesial temporal sclerosis (Grade 3) | Left anterior temporal lobe | 30 | M |
| E219 | Epilepsy, reduced hippocampal neuronal density | Right temporal lobe | 51 | F |
| T180 | High grade glioma | Left frontal lobe | 52 | F |

Supplementary table 3: Case details for the post-mortem control tissue used to culture pericytes in this study

| **Case** | **Cause of death** | **Post-mortem delay (hours)** | **Age (years)** | **Sex** |
| --- | --- | --- | --- | --- |
| H189 | Asphyxia | 16 | 41 | M |
| H238 | Dissecting aortic aneurysm | 16 | 63 | F |
| H239 | Ischemic heart disease | 15.5 | 64 | M |
| H244 | Ischemic Heart disease – coronary atherosclerosis | 16 | 76 | M |

Supplementary table 4: Case details for the post-mortem Parkinson’s disease tissue used to culture pericytes in this study

| **Case** | **Cause of death** | **Post-mortem delay (hours)** | **Age (years)** | **Sex** |
| --- | --- | --- | --- | --- |
| PD52 | Myocardial infarction | 5 | 84 | M |
| PD65 | Parkinson’s disease aspiration | 2.25 | 67 | M |
| PD71 | Pneumonia | 5.5 | 80 | M |
| PD78 | Parkinson’s disease | 5.5 | 80 | M |

Supplementary table 5: CBA kits used in this study

| **Antibody** | **Cat no.** | **Bead position** |
| --- | --- | --- |
| sCD54/ICAM-1 (intracellular adhesion molecule-1) | 560269 | A4 |
| sCD106/VCAM-1 | 560427 | D6 |
| Fractalkine (CX3CL1) | 560265 | C6 |
| C-CSF (granulocyte-colony stimulating factor) | 558326 | C8 |
| GM-CSF (granulocyte macrophage-colony stimulating factor) | 558335 | C9 |
| IL-6 (interleukin-6) | 558276 | A7 |
| IL-8 (interleukin-8) | 558277 | A9 |
| IP-10 (interferon gamma-induced protein 10) | 558280 | B5 |
| MCP-1 (monocyte chemoattractant protein-1) | 558287 | D8 |
| RANTES (regulated on activation, normal T cell expressed and secreted) | 558324 | D4 |

Supplementary table 6: Toll-like receptor ligands

| **TLR ligand** | **Concentration** | **Supplier** | **Catalogue** |
| --- | --- | --- | --- |
| Imuquimod | 5 µg/mL | Invitrogen | Tlr-imiq |
| LPS | 10 ng/mL | Sigma | L4391 |
| Poly(I:C) | 5 µg/mL | Invivogen | Tlr-pic |
| Pam3CSK4 | 300 ng/mL | EMC Biochemicals | L2000 |
| Pam2CSK4 | 100 ng/mL | EMC Biochemicals | L2020 |
| CPG ODN-2395 | 1 µM | Invivogen | Tlr-hodnb |
| Flagellin | 10 ng/mL | FLA-ultrapure | Tlrl-pafla |

Supplementary table 7: Studies demonstrating cell type, species, concentration of α-syn, endotoxin contamination present and whether α-syn induced inflammation

| **Study** | **Species** | **Cell type** | **α-synuclein source** | **α-synuclein concentration** | **Endotoxins present (Endotoxin units)** | **Inflammation** |
| --- | --- | --- | --- | --- | --- | --- |
| White et al. (2018) | Human | Peripheral blood mononuclear cells (PMBCs) | Bacterial expression in lab | 2 nM | 0.02-1.3 EU/mL | ✓ |
| Su et al (2009) | Rat | Primary microglia | Bacterial expression in lab | 2.5 – 10 nM | 0.06 EU | ✓ |
| Beraud et al (2011) | Mouse | BV-2 microglia and Primary microglia P1-P3 mice | Bacterial expression in lab | 50 nM | 0.13 EU | ✓ |
| Reynolds et al (2007) | Mouse | Primary microglia P-1-P2 | Bacterial expression in lab | 100 nM | <0.05 EU | ✓ |
| Zhang et al (2005) | Rat | Primary microglia | r-Peptide | 25 – 250 nM | <1.3 EU/mg | ✓ |
| Couch et al (2011) | Mouse | BV-2 | r-Peptide | 3 µg (208 nM) | Did not test | ✓ |
| Cao et al (2012) | Mouse | Primary microglia P0 – P3 | r-Peptide | 500 nM | Did not test | ✓ |
| Grozdanov et al (2019) | Mouse and Human | Human blood monocytes, mouse BV-2 microglia | Bacterial expression in lab | 10µg/mL (694 nM) | <0.02 EU/µg protein | ✓ |
| Fellner et al (2013) | Mouse | Primary microglia P1 – P3 | Bacterial expression in lab | 3µ M | <1 EU/mg | ✓ |
| Dohgu et al (2019) | Rat | Primary pericytes | Bacterial expression in lab | 3.5 µM | Did not test | ✓ |
| Hoenen et al (2016) | Mouse | Primary microglia | Bacterial expression in lab | 5 µM | Tested but not shown | ✓ |
| Lee et al (2010) | Rat | Primary microglia P1 – P2 | r-Peptide | 10 µM | <1 EU/mg | ✓ |
| Hughes et al (2019) | Mouse | BV-2 microglia | Bacterial expression in lab | 10pM – 100 µM | <0.006 ng/mL | ✓ |
| Li et al (2020) | Mouse | Primary microglia | SinoBiological | 50 – 250 nM | - | **✕** |
| Russ et al (2021) | Human | IPSC-derived astrocytes | Bacterial expression in lab | 10 µM | - | ✓ |
|  |  |  |  |  |  |  |
